# Research overview of ethnic medicines for the treatment of rheumatoid arthritis

**DOI:** 10.3389/fphar.2025.1662130

**Published:** 2025-09-23

**Authors:** Xiao-jing Lei, Hong-mei Sun, Yun-xia Qu, Qing-zhu Xu, Ying Zhou, Huai Xiao, Li-juan Li, Zhi-bin Yang

**Affiliations:** ^1^ Yunnan Provincial Key Laboratory of Entomological Biopharmaceutical R&D, College of Pharmacy, Dali University, Dali, Yunnan, China; ^2^ College of pharmacy, National-Local Joint Engineering Research Center of Entomoceutics, Dali, Yunnan, China; ^3^ School of Clinical Medicine, Dali University, Dali, Yunnan, China

**Keywords:** rheumatoid arthritis (RA), traditional Chinese medicine, ethnomedicine, ethnic medicine, review

## Abstract

Rheumatoid arthritis (RA) is a common clinical autoimmune disease, characterized by persistent synovitis, cartilage destruction and systemic complications, and the incidence of which is increasing every year. The current study found that the characteristics of multi-component, multi-target, and overall regulation of ethnic drugs have significant effects on the prevention and treatment of RA, with less toxicity and side effects. However, there is a lack of comprehensive overview of existing ethnic medicine treatments for RA. This paper summarizes the available ethnomedicines for RA and reviews the current status of research on ethnomedicines against RA, experimental and clinical studies on some common ethnomedicines for RA, and the effects of non-pharmacological treatments on RA. First of all, the search terms “Chinese medicine,” “traditional Chinese medicine,” “ethnomedicine,” “arthritis,” “rheumatoid arthritis,” “Mongolian medicine,” “Zhuang medicine,” “Miao medicine” and “Tibetan medicine” were used as search terms to search for relevant literature using Pubmed, China National Knowledge Internet (CNKI) and Wanfang data (1996–2024); secondly, the missing basic information of ethnomedicine collected was supplemented using two databases, namely, Clinical Diagnosis and Treatment Knowledge Base and the Basic Information Base of Chinese Herbal Medicine-Yaozhi data. Among the national medicines for the treatment of RA, the Tibetan medicine, Miao medicine, Zhuang medicine, Mongolian medicine, Dai medicine and Hui medicine are mostly used in the majority of medicinal materials, and the research reports are also in the majority. Other national medicines are also available, but the medicinal materials used are less, the research reports are less, or even not. The treatment of RA, whether experimental or clinical research, is mainly based on compound prescriptions, less use of single herbs, and non-drug therapy. This may provide drug selection and insights for the future experimental and clinical research directions of ethnic drugs widely used in the treatment of RA. Nevertheless, there is still a need for more mechanism research and exploration, as well as pharmacological, toxicological and clinical research on existing or untapped ethnomedicines.

## 1 Introduction

Arthritis, an inflammatory condition affecting the joints and surrounding tissues, can be attributed to various factors such as inflammation, infection, degeneration, trauma, or other etiological agents (Di Matteo et al., 2023). It is commonly categorized into osteoarthritis, rheumatoid arthritis, infectious arthritis, metabolic arthritis, and juvenile idiopathic arthritis based on its underlying cause. Rheumatoid arthritis (RA) is widely recognized in contemporary medicine as a prevalent clinical autoimmune disorder, distinguished by the infiltration of inflammatory cells in the synovial joint cavity and the occurrence of bone erosion caused by vascular opacities (Zhang et al., 2023). In severe instances, this condition can result in joint deformity, limited mobility, functional impairment, and significant disability. Nevertheless, the etiology of this disease remains elusive, with prevailing consensus suggesting a potential correlation between its onset and genetic and environmental influences (Ye et al., 2022).

In the realm of traditional medicine, the pathogenesis of RA is typically categorized into two components, which are attributed to the influence of wind, cold, and damp evil, with wind being the most persistent among all ailments (Ma and Gan, 2025). Consequently, the manifestation of symptoms such as joint pain is commonly observed. The internal cause is primarily attributed to kidney deficiency, as the kidney serves as the innate foundation responsible for storing the essence and healthy qi of the human body (Zhao et al., 2011). In the event of kidney weakness, various diseases may arise. Insufficient kidney yang results in a lack of qi source, leading to laxity in the interspaces of the skin and muscles, making the body more susceptible to malevolent qi (Xu D. et al., 2018).

Ethnic medicine offers alternative perspectives on RA. Hui medicine believes that the decay of the body’s endowment and abnormal body fluids are responsible for rheumatic diseases. Mongolian medicine attributes RA to an increase in “Xieriwusu (yellow liquid),” a struggle with “Badagan (cat liquid)” and blood, which condenses in the joints and obstructs the flow of qi and blood (Zhou X. P. et al., 2017). Zhuang medicine suggests that various factors weaken the body, allowing wind, dampness, cold, and heat toxins to invade. These pathogenic toxins block tendons, bones, and muscles while obstructing the three channels and two pathways, leading to asynchrony of the three qi and causing RA (Zhou J. et al., 2017; Xu H. et al., 2018).

**FIGURE 1:**
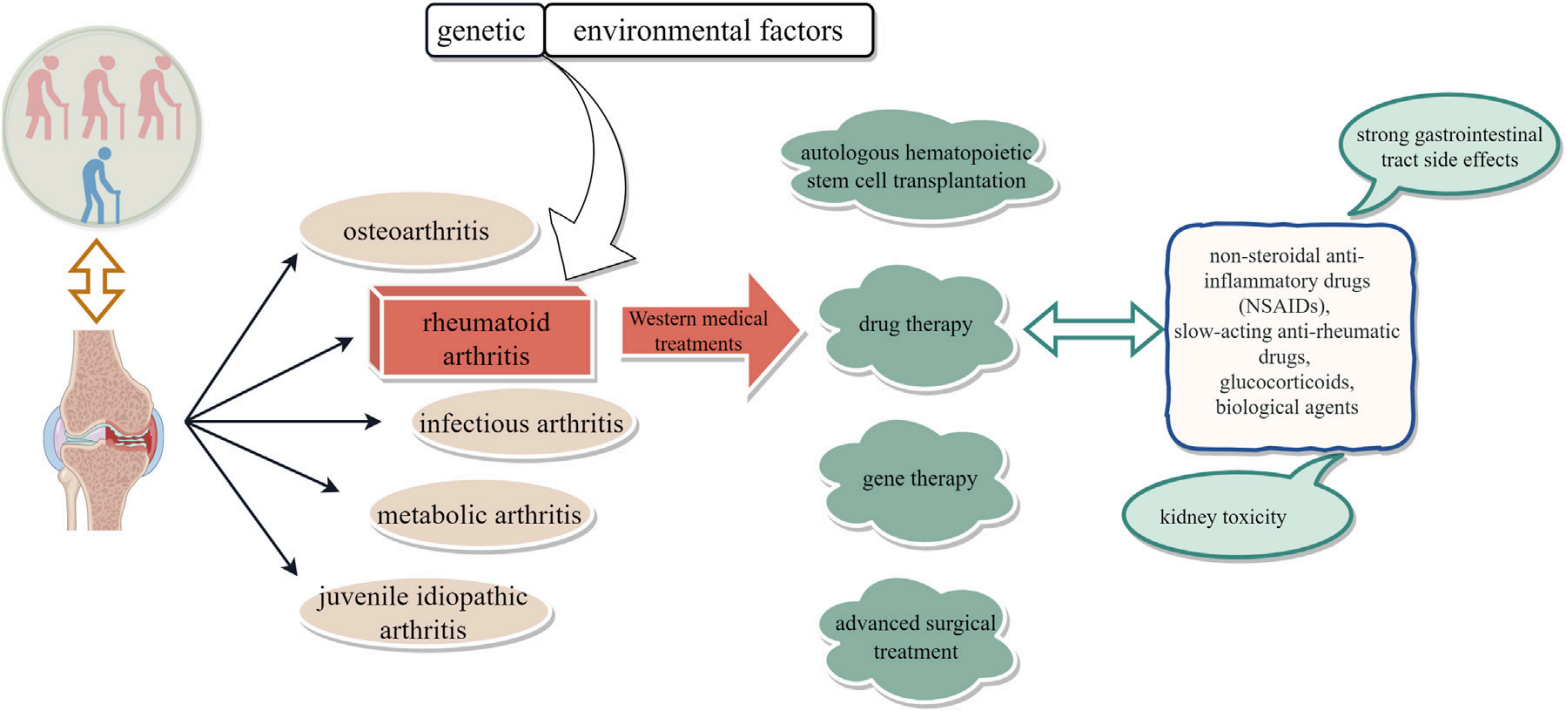
Outline of Western therapies for rheumatoid arthritis.

RA, characterized by an unclear etiology, a protracted disease course, and a high disability rate, remains one of the chronic refractory diseases. It affects approximately 0.5%–1% of individuals in Western countries and 0.2%–0.9% of the Chinese population (Li et al., 2025). RA can manifest at any age but is more prevalent among women (Jakobsson et al., 2022). With its disability rate ranking second only to diseases like malaria, RA poses a significant threat to patients’ work capacity, overall health, and quality of life. Currently, there is no definitive cure for RA; treatment strategies primarily focus on controlling joint damage and functional impairment, alleviating symptoms, and enhancing quality of life (Cush, 2022). Conventional Western medical approaches encompass drug therapy (such as non-steroidal anti-inflammatory drugs [NSAIDs], slow-acting anti-rheumatic drugs [SAARDs], glucocorticoids, and biological agents), autologous hematopoietic stem cell transplantation, gene therapy as well as advanced surgical interventions (Gaffo et al., 2006; Abbasi et al., 2019); however, their efficacy remains limited. Therefore, the urgent need to identify efficacious yet low-toxicity therapeutic agents for RA persists (Figure 1).

Chinese medicine is the general term for all ethnic medicine in China, including Chinese and minority medicine. Approximately 75% of Chinese individuals and those of Chinese descent opt for traditional Chinese medicine (TCM) and other complementary therapies to address arthritis and musculoskeletal disorders (Pan et al., 2019). In recent years, TCM and ethnomedicine have demonstrated remarkable efficacy in the prevention and treatment of metabolic diseases such as RA (Wang Y. et al., 2021; Zhang et al., 2019; Jakobsson et al., 2022; Wang et al., 2022; Wu et al., 2022).

Ethnic medicine refers to the medicinal practices employed by ethnic minorities, which are guided by traditional medical theories and practices (Zhou X. Y. et al., 2024). A subset of alternative medicine, minority medicine incorporates the distinctive climate and diet of local ethnic groups, providing unique insights into the treatment of RA. These ethnic medicines have shown exceptional clinical efficacy and advantages in managing RA, underscoring the crucial role played by ethnomedicine in safeguarding human health and wellbeing (Jiang et al., 2023).

Therefore, this study aims to review recent domestic and international literature from databases including PubMed, CNKI, and Wanfang Data to summarize currently available ethnic medicines for RA treatment. Categorized by ethnic groups, selected common ethnic medicines for RA will be examined regarding their potential in RA treatment through experimental studies, clinical research, and non-pharmacological therapies. This work seeks to address the current gap in comprehensive summaries of ethnic medicines, with the goal of providing references for rational medication use and research directions for future experimental and clinical studies on RA.

## 2 Methodology

The scientific electronic databases Pubmed, China National Knowledge Internet (CNKI), and Wanfang Data were searched, and research and review articles were extracted from the above databases. All data were screened until 2024 (1996–2024). Various keywords and topics closely related to systematic reviews were used for retrieval, such as “Chinese medicine,” “traditional Chinese medicine,” “ethnic medicine,” “arthritis,” “rheumatoid arthritis,” “Mongolian medicine,” “Zhuang medicine,” “Miao medicine,” “Tibetan medicine,” “traditional Chinese medicine for arthritis,” “ethnic medicine for arthritis,” and “Tibetan medicine for arthritis,” etc. The classification and collection are primarily based on ethnic groups, with the most common and extensively studied ethnic medicines selected for description from each ethnic group. Inclusion criteria: a) Research articles and patents on the use of ethnic medicine to treat RA. b) Full-text research, research reports, and monographs on the use of ethnic medicine or ethnic medicine pathways to treat RA. c) Human clinical trials and basic research on the use of ethnic medicine to improve RA. Exclusion criteria: a) Articles or studies that review treatment pathways without clear methodology and do not explicitly state which specific ethnic group the ethnic medicine originates from. b) Duplicate studies. c) Abstracts or draft manuscripts only. d) Lack of transparent methodology and objectives.

First of all, preliminary data collection, collation, and analysis. Secondly, the Clinical Diagnosis and Treatment Knowledge Base (http://lczl.med.wanfangdata.com.cn/) and the Basic Information Base of Chinese Herbal Medicine-Yaozhi data (https://db.yaozh.com/zhongyaocai) are used to supplement the basic information of the missing data of the medicinal materials for the treatment of rheumatoid arthritis by ethnic medicines, as the Supplementary Material of this paper (You can find it in the Supplementary Material that we have compiled for this article.).

## 3 The pathogenesis and etiology of RA

**FIGURE 2:**
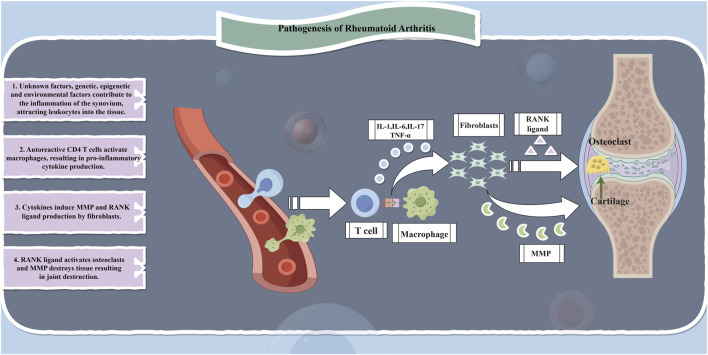
Pathogenesis of rheumatoid arthritis.

RA is a chronic, inflammatory, systemic autoimmune disease that affects approximately 5–10 individuals per 1,000 population. The main feature of RA is non-suppurative inflammation of joints and joint tissues, primarily manifested as synovitis leading to damage in cartilage, ligaments, tendons, and other joint tissues as well as multi-organ impairment. The underlying pathological changes involve synovitis with acute swelling and exudation, chronic granulocytic infiltration, synovial hyperplasia, and vasculitis (Weddell and Hider, 2021; Zhang L. et al., 2021). Similar to numerous autoimmune diseases, the etiology of RA is multifactorial involving genetic factors accounting for 50% of the risk (Giannini et al., 2020; Scherer et al., 2020; Testa et al., 2021). Whereas the cascade of innate and adaptive immune responses plays a crucial role in the inflammatory process in RA; it is driven by inflammatory cytokines and autoantibodies which are sustained by epigenetic alterations in fibroblast-like synoviocytes further promoting inflammation development. During this period, various immune cells infiltrate into the synovium and body fluids resulting in an extensive release of cytokines, chemokines, and autoantibodies along with reactive oxidative stress (ROS) within the synovium and its interstitial spaces ultimately leading to joint destruction (Zhang Z. J. et al., 2021; Chen et al., 2019). Consequently, studies related to RA treatment have predominantly focused on immune mechanisms including immune cells, cytokines, TNF-α inhibition, chemokines, interferons, and JAK/STAT signaling pathways while also partially considering enzymes, inflammatory mediators, and other associated factors along with alternative pathways (Figure 2).

## 4 Limitations of Western medicine in the management of RA

RA is a multifactorial disease with an unclear etiology. Without systematic treatment, it can lead to recurrent symptoms eventual joint deformity, and loss of function. Therefore, the treatment of RA has two main objectives: symptom relief and maintenance of function, as well as slowing down tissue damage progression. Western medical treatments include general measures such as rest, joint braking, functional exercise, physical therapy, and bed rest; drug therapy including non-steroidal anti-inflammatory drugs (NSAIDs), disease-modifying antirheumatic drugs (DMARDs), biological agents and glucocorticoids (GCs); autologous hematopoietic stem cell transplantation; gene therapy; and surgical intervention. Among these options, drug therapy is the most important approach for managing RA symptoms (Zhang L. et al., 2021; Gaffo et al., 2006). NSAIDs are widely used but have strong gastrointestinal tract side effects and kidney toxicity with long-term use; DMARDs are the first-line drugs for clinical diagnosis but almost all can cause drug-induced liver injury; GCs are reserved for patients who exhibit intolerance to other RA medications, yet their potential to induce osteoporosis at higher doses and other associated adverse effects remains a concern. Based on the current understanding of the immune mechanisms of RA that has led to an increasing number of novel biological agents being used to treat RA. For instance, TNF-α antagonists such as etanercept (Enbrel), infliximab (Remicade), adalimumab (Humira), certolizumab-pegol (Cimzia), and golimumab (Simponi) (Lim et al., 2018; Tang, 2019). Despite their enhanced clinical efficacy, these aforementioned biological agents incur substantial treatment costs, thereby exacerbating the financial burden experienced by patients. However, autologous stem cell transplantation is still being investigated as a treatment option, with a high recurrence rate observed. The safety profile of gene therapy remains unknown and carries inherent risks. Although surgical intervention can enhance joint function, it often necessitates postoperative drug maintenance therapy. Given the chronic nature of RA, it is evident that Western medicine’s treatment modalities have certain limitations, highlighting the urgent need for novel therapeutic drugs or approaches to address RA.

## 5 Advantages of ethnic medicine in the treatment of RA

The medicine of ethnic minorities constitutes a significant component of TCM. Throughout its development, it has been characterized by the “unity of diversity and unity of difference,” representing a medical science that has gradually evolved and continuously enriched over thousands of years through the production, living practices, and struggles against diseases by the Chinese people. The government attaches great importance to the development of ethnic medicine. In the White Paper on *Traditional Chinese Medicine* in China, clear definitions have been provided regarding the inherent relationship between ethnic minority medicine and Chinese medicine, as well as highlighting the crucial status of Chinese medicine along with development guidelines and support measures. These provisions serve as an essential guarantee for promoting ethnic minority medicine’s progress (Liang, 2022). Policies and regulations such as *The Law of People’s Republic of China on Traditional Chinese Medicine, Outline Strategic Plan for Development (2016–2030), and The 13th Five-Year Plan for Promoting Development in Ethnic Areas and Less Populated Ethnic Minorities* emphasize developing ethnic medicine as a key task (Gao et al., 2020a). With increasing funding from provincial governments and national fund projects each year, approximately 70% of medicinal materials used are derived from ethnic sources nationwide. Moreover, there exist 116 enterprises specializing in producing ethnically-based drugs encompassing a total variety count reaching 4317 types. Additionally, there are 137 national hospitals offering preparations based on national medicines totaling 3910 varieties available to patients seeking treatment options within this domain. Analyzing patent applications related to ethnic medicines’ trends over time reveals they have maintained relatively stable growth rates. Between 1994 and 2021 alone accounted for issuing approximately 97% of patents associated with these specific medicinal practices (Dai et al., 2022). Recent years have witnessed a significant emphasis on the advancement of ethnic medicine in the country, with researchers increasingly driven to explore novel biologically active compounds derived from this field. Ethnic medicine holds immense research value and has paved the way for a new therapeutic approach towards diseases.

Conventional therapeutic drugs are not the optimal choice for treating RA. The “dialectical treatment” ideology of traditional Chinese medicine, which combines the internal or external use of Chinese medicine with acupuncture, moxibustion, tuina, and other special treatments, has been widely used in clinical practice and exhibits remarkable efficacy, low recurrence rates, and minimal toxic side effects. Ethnic medicines such as Mongolian medicine, Tibetan medicine, Hui medicine, Zhuang medicine, and Dai medicine have also demonstrated similar curative effects through long-term clinical practice and accumulation. With an increasing number of researchers discovering new bioactive compounds from ethnic medicines, studies on their effectiveness in treating RA have shown that compared to Western medical treatment options they can significantly reduce inflammatory responses; alleviate joint swelling; inhibit synovial tissue proliferation; improve RA treatment efficacy; shorten treatment duration; reduce drug resistance and alleviate symptoms (Wu et al., 2021; He et al., 2022; Adorisio et al., 2019; Zhou et al., 2022; Lan et al., 2015; Yang et al., 2021). Comprehensive evaluation indicates that ethnic medicines offer certain advantages over conventional therapies for treating RA while also possessing greater development potential and research value.

## 6 Ethnomedicine-based approaches for RA treatment

**FIGURE 3:**
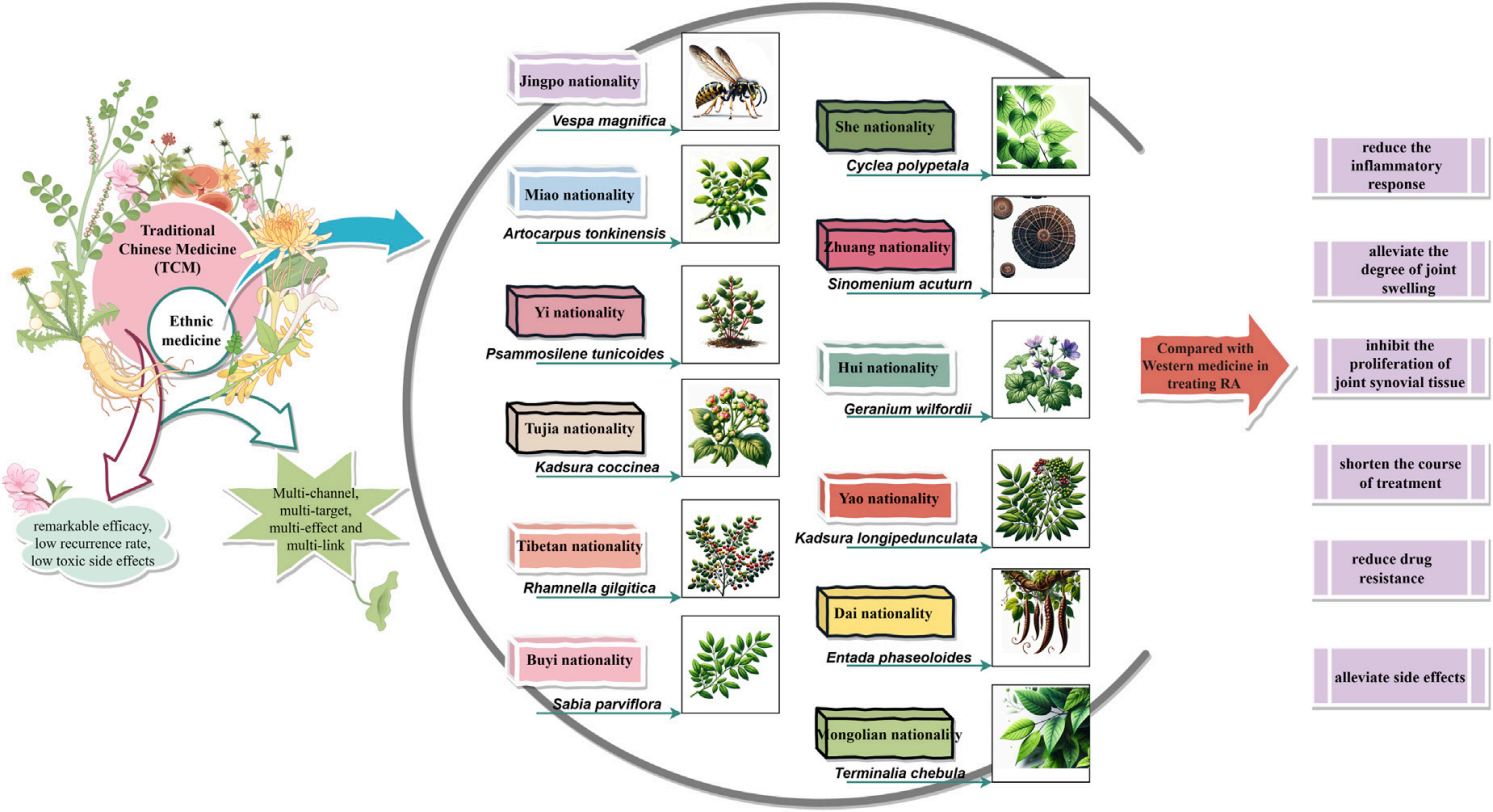
Summary of the therapeutic advantages of ethnomedicine.

Ethnic medicine has a rich history dating back to the Shang Dynasty, and its inheritance and development have led to the emergence of numerous renowned herbal works that have played a crucial role in supporting and promoting ethnic medicine’s growth and drug usage in ethnic regions. As such, ethnic medicine therapies, including monomers, classical compound prescriptions, and non-pharmacological treatments, have become widely recognized by patients due to their multi-component nature, multi-target effects, and overall regulation characteristics that provide unique clinical efficacy for treating RA. In this paper, we summarize current medicinal materials (prescriptions) used by various ethnic groups for treating RA based on relevant data and literature reviews (see Supplementary Table S1). In addition, we discuss experimental research findings and clinical studies conducted by scholars at home and abroad regarding common ethnic medicines’ effectiveness in treating RA (Figure 3).

### 6.1 Experimental research against RA in ethnic medicine

In recent years, numerous studies have been conducted by scholars both domestically and internationally on the anti-RA properties of ethnic medicine. Ethnic medicine has demonstrated considerable efficacy in reducing the inflammatory response, as well as exhibiting analgesic and anti-inflammatory effects. These effects effectively enhance the prevention and progression of RA, while also mitigating a range of complications associated with the condition, thereby showcasing superior therapeutic advantages.

#### 6.1.1 Experimental research on single ethnic medicine

Currently, the most extensively investigated single ethnic medicine materials for anti-RA include *Vespa magnifica* (Smith), *Artocarpus tonkinensis* (A.Chev. Ex Gagnep), *Psammosilene tunicoides* (W.C.Wu et C.Y.Wu), *Kadsura coccinea* (K. coccinea), *Rhamnella gilgitica* Mansf. et Melch, *Sabia parviflora* Wall. ex Roxb., *Cyclea polypetala* Dunn, *Sinomenium acuturn* (Thunb.) Rehd. et Wils, *Geranium wilfordii* Maxim., *Kadsura longipedunculata* Finet et Gagnep., *Entada phaseoloides* (L.) Merr., and *Terminalia chebula* Retz. These substances have shown significant therapeutic efficacy in collagen-induced arthritis rat (mice) models or *in vitro* models of RA-associated inflammation.

##### 6.1.1.1 Jingpo medicine

The Jingpo people have a special treatment for RA called wasp venom therapy (Duan et al., 2020). Therefore, later researchers have investigated the pharmacological effects of wasp treatment of RA. The ancient Chinese Jingpo national minority prescribed wasp venom (WV, *V. magnifica*, Smith), an insect secretion, to treat RA. Ni Lianli along with colleagues looked over what was the most effective anti-RA portion of WV-I (molecular weight less than 3 kDa), WV-II (molecular weight 3–10 kDa), and WV-III (molecular weight more than 10 kDa) that were separated from WV (Ni et al., 2023; Ni, 2021). Additionally, they investigated the molecular mechanism of WV and WV-II, which were the most effective components in RA. WV has been linked to oxidation-reduction, inflammation, and apoptosis, according to an analysis of its RNA using RNA sequencing. The outcomes demonstrated that in comparison to the WV-I treatment group, WV, WV-II, and WV-III significantly decreased the number of cells in the human MH7A cell line. All the same, compared to the IL-6-induced group, WV-III did not significantly lower the quantity of STAT3 luciferase activity. They picked WV and WV-II to investigate their anti-RA mechanism further since it had been previously reported that WV-III included significant allergens. Furthermore, by blocking the JAK/STAT signaling pathway, WV and WV-II reduced the amounts of IL-1β and IL-6 in TNF-induced MH7A cells. On top of that, WV and WV-II inhibited TrxR function to generate ROS and trigger cell death. Lipid ROS accumulation by WV and WV-II may lead to GPX4-mediated ferroptosis. When combined, WV and WV-II were able to affect MH7A cells’ redox homeostasis, ferroptosis, and JAK/STAT signaling pathways, making them promising RA treatment agents. In addition, Gao et al. (2020b) investigated the ameliorative effect of wasp venom on collagen-induced arthritis (CIA) in rats, evaluating its inhibitory impact on inflammation and autoimmune responses. The results showed that various doses of wasp venom significantly alleviated paw swelling, arthritis scores, histopathological scores of ankle synovial tissue lesions, synovial cell proliferation, and inflammatory cell infiltration compared to the untreated control group. In the aspect of immune regulation, wasp venom reduced immune serum globulin levels and suppressed Th cell-mediated immune response while notably enhancing Tc cell and Treg cell function in spleen cells. Furthermore, wasp venom’s suppression of interleukin-1β (IL-1β), tumor necrosis factor-α (TNF-α), interleukin-8 (IL-8), interleukin-6 (IL-6), cyclooxygenase-2 (COX-2) and prostaglandin E2 (PGE2) levels in rat serum was in line with its immunosuppressive action. These results suggest that wasp venom exhibits anti-arthritis activity potentially attributed to its inhibitory effects on immune regulation and inflammation.

##### 6.1.1.2 Miao medicine

The Miao ethnic group employs *A. tonkinensis* (A.Chev. Ex Gagnep) as a traditional medicinal plant to treat RA (Orecchini et al., 2021). Consequently, subsequent generations have investigated the pharmacological effects of *A. tonkinensis* in the treatment of RA. In an experimental investigation of the anti-RA properties of *A. tonkinensis* leaf decoction, Adorisio et al. (2019) discovered that it significantly alleviates RA symptoms in mice. Through histological evaluation, analysis of autoimmune gene expression profile, lymphocyte activation, participation of lymphocyte subsets, cytokine production during Th17 cell differentiation, and other experiments, they discovered that its primary mode of action is to prevent Th17 cells, which are thought to play a role in the development of arthritis, from maturing and from functioning. Moreover, *P. tunicoides* is also a medicine used by Miao nationality and Yi nationality to treat RA (He et al., 2021a; Zhou L. et al., 2024; Wei et al., 2022), and its pharmacological effects have been researched by subsequent researchers. The impact of *P. tunicoides* total saponins on fibroblast-like synovial cells in RA was examined by He et al. (2021b). The MTS colorimetric test, western blotting, immunofluorescence method, and q-PCR were among the experimental techniques used to assess the expression of the concentrations of NLRP3, ASC, and Caspase-1 mRNA and protein. Additionally, an ELISA kit and q-PCR were used to quantify the release of the inflammatory factors IL-1β, IL-18, and IL-6 as well as the matching mRNA level. The findings revealed that *P. tunicoides* total saponins effectively suppress the activation of NLRP3 inflammasomes by inhibiting proliferation and migration while inducing apoptosis in MH7A cells.

##### 6.1.1.3 Tujia medicine and Tibetan medicine

*Kadsura coccinea* is a medicinal plant traditionally used by the Tujia ethnic group for RA (Yang Y. P. et al., 2022; Yang L. et al., 2022), through the anti-RA-FLS inquiry, Yang et al. (2021) evaluated the effect of triterpene components in *K. coccinea* on inflammatory cytokines IL-6 and TNF-α levels in LPS-induced RAW 264.7 macrophages. The results revealed significant inhibition of IL-6 release by certain compounds, with IC_50_ values of 8.15 and 9.86 μM, respectively. In addition, some compounds exhibited notable suppression of RA-FLS cell proliferation *in vitro*.

*Rhamnella gilgitica* Mansf. et Melch (Shengdeng) is a medication commonly employed in Tibetan traditional medicine for RA (Zou et al., 2020; Fan, 2023; Wang et al., 2021b; Li, 2020; Pei, 2015), the anti-RA effect of Shengdeng on CIA in rats and its mechanism were investigated by Su Jinsong et al. (Su et al., 2021). Treatment with Shengdeng was observed to inhibit the arthritis index and swelling of the foot, reduce inflammation and synovial hyperplasia in the synovial tissue of the knee joint, suppress bone erosion, decrease levels of cytokines such as TNF-α, IL-1β, IL-6, IL-17 and Interferon-γ (INF-γ), increase the expression of SOCS1 and SOCS3 in both protein and mRNA and inhibit the expression of JAK2 and STAT3 in both protein and mRNA. Additionally, upregulation of Bax and Caspase3 expression along with downregulation of Bcl-2 expression were noted. These results imply that the prevention of inflammatory responses, stimulation of cell death, and modification of the JAK-STAT pathway may all be responsible for the anti-RA impact.

##### 6.1.1.4 Buyi medicine

The medicinal plant *S. parviflora* Wall. ex Roxb., traditionally used by the Buyi ethnic groups for RA (Yang Y. et al., 2022; Xu X. Y. et al., 2018; Wang M. L. et al., 2018), was investigated by Zhou et al. (2022) to identify its active leaf ingredients and evaluate their effects on rats with RA. The study demonstrated that the leaf extract exhibited anti-inflammatory properties by reducing foot swelling in RA rats, decreasing thymus and spleen indexes, and preventing the release of inflammatory substances from the serum of RA rats, including TNF-α, IL-1β, IL-6, IL-10, IL-15, and vascular endothelial growth factor (VEGF). Moreover, It successfully prevented the activation of TRP protein transient receptor potential melastatin-5 (TRPM-5) and transient receptor potential channel-6 (TRPC-6), as well as nuclear factor kappa-Bp65 (NF-κBp65), an inflammatory protein. The presence of significant bioactive compounds in the leaves of *S. parviflora* Wall. ex Roxb. has been confirmed, suggesting their potential role in RA treatment through inhibition of inflammatory factor release and involvement in the NF-κB pathway as well as modulation of inflammatory protein expression within the TRP protein family.

##### 6.1.1.5 She medicine and Zhuang medicine

The medicinal use of *C. polypetala* Dunn is prevalent among the She nationality (Sun, 2018; Zhang Y. Q. et al., 2018; Mo et al., 2017), and succeeding generations have researched its pharmaceutical properties. Xu (2018) discovered significant anti-RA activity in the ethyl acetate fraction of *C. polypetala* Dunn through animal model screening. Subsequent investigations employing ELISA, Western-blot, and RT-PCR techniques were conducted to further elucidate the molecular mechanism of action exhibited by the ethyl acetate fraction, revealing its potential role in inhibiting RA occurrence and development by modulating the TLR4/NF-κB signal transduction pathway.

*Sinomenium acuturn* (Thunb.) Rehd.et Wils. is a medicine used by the Zhuang nationality for RA (Cao et al., 2020; Jia et al., 2024; Yu et al., 2022), the effect of total alkaloids of *S. acuturn* on platelet endothelial cell adhesion molecule 1 (PECAM-1) and P-selectin (CD62P) expression in the serum of rats with RA was investigated by Cao et al. (2022). The levels of transcription of PECAM-1 and P-selectin in the serum of RA model rats may be decreased by sinomenine, which is an effective treatment for RA.

##### 6.1.1.6 Hui medicine and Yao medicine

The Hui people utilize *G. wilfordii* Maxim. as a medical treatment for RA (Zhou X. P. et al., 2017), Yang X. S. et al. (2022) explored the effect of *G. wilfordii* cataplasm on RA rats and found that it had a better anti-RA effect. The foot swelling degree of rats was significantly reduced, the pathological changes of ankle joints were improved to different degrees, and the levels of IL-6, TNF-α, VEGF-A, and IL-17 in serum were significantly reduced.

Yao nationality makes use of a medication called *K. longipedunculata* Finet et Gagnep. for RA (Tang et al., 2022; Huang et al., 2022),and subsequent generations have researched its medicinal properties (Tang, 2021). To investigate the anti-RA effect and associated mechanism of action of *K. longipedunculata* Finet et Gagnep ethyl acetate extract, Qin et al. (2017) constructed CIA model rats. It was found that ethyl acetate extract of *K. longipedunculata* Finet et Gagnep. could significantly inhibit foot swelling in CIA model rats and reduce the levels of IL-1β, IL-6, TNF-α of the serum and NF-κBp65 mRNA levels of synovial tissue. Compared with Tripterygium wilfordii and dexamethasone, the effect was stronger than that of Tripterygium wilfordii and weaker than that of dexamethasone.

##### 6.1.1.7 Dai medicine and Mongolian medicine

The Dai nationalities take advantage of *E. phaseoloides* (L.) Merr. as a medication for RA (Xiong et al., 2021; Xu et al., 2021; Xiong, et al., 2018), Xu et al. (2021) probed into the therapeutic effect of *E. phaseoloides* (L.) Merr. extract on bovine type II collagen-induced RA model rats. The outcome demonstrated that the *E. phaseoloides* (L.) Merr. the extract could lower the spleen index, decrease the degree of edema in the paws and ankle joints of CIA rats, and downregulate the levels of IL-1β, IL-17, and PGE2 in the serum. It significantly alleviated the proliferation of synovium, decreased the production of pannus and the infiltration of inflammatory cells, and improved the pathological damage of cartilage lesions. On the other hand, the outcomes of *in vitro* cell tests suggest that *E. phaseoloides* (L.) Merr. extract may have anti-inflammatory properties by preventing the expression of inflammatory factors such as TNF-α, PGE2, and IL-1β.

A medication of treating RA taken by people of Mongolian descent is *T. chebula* Retz. (Zhao et al., 2023; Liu and Dong, 2023; He X. Y. et al., 2021; Lui, 2022), Liu et al. (2020) probed into the effect of *T. chebula* Retz. water extract on intestinal immune regulation in collagen-induced arthritis rats. It was discovered that the aqueous extract of *T. chebula* Retz. could control the imbalance of intestinal flora, successfully lower the expression of serum CD4 and CD25 in CIA rats, and considerably improve the arthritis performance of CIA model rats. This may be one of the effective mechanisms of *T. chebula* Retz. for the treatment of RA in clinical practice.

#### 6.1.2 Experimental research on the compound prescription of ethnic medicine

The treatment of RA in ethnic medicine primarily relies on compound prescriptions, which combine various components to not only mitigate the side effects associated with individual drugs but also enhance their efficacy. This approach enables a multi-targeted, multi-level, and multi-path comprehensive therapeutic effect of the compound prescriptions.

##### 6.1.2.1 Tibetan medicine

Li Z. et al. (2022) conducted a study to examine the correlation between the chemical crosstalk interaction of Tibetan medicine twenty-five wei’er tea pills (TFP) (Zhang et al., 2022; Zhang, 2017; Huang, 2001) and the gut microbiota and its host. The researchers employed non-targeted metabolomics and the 16S rRNA sequencing to analyze CIA rats. The results revealed that after receiving TFP treatment, the joint pathological alterations in the CIA model rats significantly improved. Additionally, TFP therapy also resulted in elevated levels of IL-4 and IL-10, as well as lower levels of TNF-α and IL-6 in the serum. It was also observed that the gut microbiome of CIA model rats was dysfunctional and the serum metabolites were changed. However, these dysfunctions and alterations were found to be reversible upon treatment with TFP. Furthermore, consistency analysis demonstrated that TFP exerted regulatory effects on various types of metabolic pathways, including the metabolism of histidine, phenylalanine, alanine, aspartic acid, glutamic acid, amino sugars, nucleotide sugars, and other metabolic pathways. Ultimately, the findings demonstrate the potential of TFP as a therapeutic agent for ameliorating RA by modulating the chemical crosstalk between intestinal flora and the host.

He et al. (2022) employed a comprehensive approach combining GC-MS, UPLC-Q-TOF/MS, network analysis, and experimental validation to elucidate the potential pharmacodynamic substances and mechanisms underlying the therapeutic effects of Wu Wei Shexiang pills (WPW) (Wencheng et al., 2020; Jiahua, 2019), a Tibetan medicine used for treating RA. The study revealed that its 11 components may serve as the primary active constituents for RA treatment. while PIK3CA, AKT, MAPK, IL-6, TNF, MMP1, MMP3, and CDK1 were identified as core targets. Signaling pathways such as PI3K-AKT and MAPK along with processes related to cell apoptosis and cell cycle regulation were found to be crucial in mediating the therapeutic effects of WPW on RA. Animal experiments and histomorphological analysis show that WPW significantly ameliorated foot swelling degree while reducing ankle joint diameter and arthritis index. it also mitigated inflammation in synovial tissue and cartilage injury in the ankle joint. Furthermore, serum levels of IL-6, IL-1β, and IL-17 were downregulated by WPW administration. According to the results of immunohistochemistry, WPW increased the expression of the Bax protein while decreasing the protein expression of AKT, PI3K, MAPK, MMP1, MMP3, CDK1, and Bcl-2.

##### 6.1.2.2 Miao medicine

In their study, Wu et al. (2021) employed data mining techniques to analyze the apoptosis and pyroptosis proteins associated with RA. In addition, they conducted molecular docking and *in vitro* experiments on key target proteins to assess the impact of the empirical formula of the Si da xue (SX) (Liang, et al., 2023; Wang, et al., 2022; Wu, et al., 2021; Yuan, et al., 2019) of Miao medicine on the proliferation, migration, and invasion of human RA-FLSs, such as MH7A cells. The wound healing experiment, transwell assay, ELISA method, and western blot method were used to assess the migration and invasion capacity, as well as the apoptosis and pyroptosis-related proteins of MH7A cells, respectively. The results showed that the prescription effectively induced apoptosis in MH7A cells, suppressed focal death, and attenuated the inflammatory response. The underlying mechanism may involve downregulation of TNF-α, IL-1β, and IL-18 cytokines, upregulation of Bax, Fas, and FasL proteins, as well as inhibition of Bcl-2 and Caspase-1 protein expression.

##### 6.1.2.3 Zhuang medicine

Yao et al. (2022) used UPLC-MS/MS metabolomics technology to elucidate the therapeutic effect of Zhuang medicine Long zuan tong bi granules (LZTBG) (Huang et al., 2019; Li et al., 2019; Yao, 2023; Chen, 2021; Tang et al., 2020) on the CIA rat model, revealing a significant inhibitory impact of LZTBG on the CIA rats. A total of 31 differential metabolites were identified, and pathway analysis mapped 11 potential efficacy-related biomarkers into the relevant pathway. These compounds encompass linoleic acid (LA), phosphatidylcholine (PC), lysophosphatidylcholine (LPC), arachidonic acid (AA), 12-HETE, α-linolenic acid (ALA), 13(S)-HOT, 2-oxobutyric acid, 3-hydroxybutyric acid, L-valine, and acetylcholine. Studies have indicated that these metabolites may modulate inflammation by regulating lipid metabolism and amino acid metabolism pathways.

##### 6.1.2.4 Mongolian medicine and Yao medicine

In addition, Zi and Yu (2015) developed models for both a chemical composition-target network and a chemical composition-target-disease network. The study revealed that Sendeng-4, a Mongolian medicine (Wang X. et al., 2018; Liu et al., 2021; Zhao et al., 2020; Xu Y. et al., 2018; Wang B. Y. et al., 2018), exhibits a multifaceted impact on diseases through the coordination of at least nine distinct targets. To validate the key targets of Sendeng-4’s chemical composition, CIA model rats were employed. Experimental findings confirmed the candidacy of TNF-α, IL-6, and PGE2 as molecular targets, thereby substantiating the anti-inflammatory properties of Sendeng-4.

Liang et al. (2019) investigated the anti-inflammatory effect of Sifangteng small compound, a Yao medicine (Fang, 2024; Luo et al., 2013), on collagen-induced RA rats. The study revealed that the Sifangteng small compound significantly attenuated acute inflammation in RA rats by targeting multiple components such as resveratrol and primarily acting on PTGS2. Moreover, it demonstrated its involvement in modulating various inflammatory immune pathways including the RA pathway and TNF pathway, ultimately exhibiting therapeutic potential for RA treatment.

##### 6.1.2.5 Tujia medicine

Tan (2011) established a murine model of arthritis induced by Freund’s complete adjuvant to investigate the therapeutic effect and mechanism of action of the Tujia national medicine compound Panax Japonicus Tablet (Song et al., 2020; Meng et al., 2019; Nan et al., 2019), in experimental arthritis treatment. The results demonstrated that Panax Japonicus Tablet inhibited joint swelling and pathological changes in mice with Freund’s complete adjuvant-induced arthritis, as evidenced by reduced arthritis index and decreased levels of pro-inflammatory cytokines IL-6, IL-8, and TNF in the serum. Furthermore, compared to the control group treated with Tripterygium wilfordii polyglycoside tablets, the treatment group exhibited superior efficacy. These findings suggest that Panax Japonicus Tablet effectively inhibits pathologic elevation of pro-inflammatory cytokines, and effectively suppresses disease progression in murine arthritis.

### 6.2 The clinical research of ethnic medicine on RA

The clinical studies of most ethnic medicines on RA primarily rely on compound prescriptions. These compound prescriptions, derived from ancient books and literature, classical prescriptions, tested formulations, and medicine pairs with anti-RA effects, have been accumulated through the clinical practices of various ethnic minorities over many years and continuously improved. They possess a comprehensive theoretical system encompassing functions such as heat and toxin clearance, dampness elimination and paralysis removal, turbidity resolution and channel dredging, as well as yin nourishment and kidney tonification. Due to their remarkable therapeutic efficacy with minimal toxicity and side effects in RA treatment, they are widely accepted by patients and extensively utilized in clinical practice. Moreover, these compound prescriptions have garnered significant attention from scholars who aim to investigate and elucidate their mechanisms of action against RA.

#### 6.2.1 Tibetan medicine

Tong luo hua shi (TLHS) is a novel formulation derived from Ding et al. (2023); Wang et al. (2021c); Wang and Lu (2020); Ding et al. (2019), a fundamental Tibetan medicine employed for the treatment of various ailments, especially RA. Liu et al. (2016) conducted a randomized, double-blind, placebo-controlled dose survey to investigate TLHS in active RA patients across five medical centers. Blood sampling, physical examination, and assessment of the American College of Rheumatology (ACR) 20% improvement criteria (ACR20) were performed biweekly pre- and post-treatment. The research showed that by adhering to ACR20 standards, TLHS effectively alleviates RA symptoms while ensuring safety. Wu wei gan lu pharmaceutical bath granules are classified as Tibetan medicine (Duo, 2018). Liu et al. (2012) used a randomized controlled approach involving 120 RA patients to evaluate the impact of these granules on clinical and laboratory indices as well as safety considerations. The findings revealed that this formula is both safe and efficacious in treating RA by significantly improving laboratory parameters and enhancing patients’ quality of life. Xiao bi ointment and Wu wei kuan jin teng decoction can also fall under the category of Tibetan medicines. Qieji and Gezhi (2021), through an observation-based study design, assessed the clinical efficacy achieved by combining these two prescriptions for treating RA among 60 randomly divided cases into two groups; pain levels and joint function scores were evaluated before and after treatment within each group respectively. Results indicated that utilizing Wu wei Kuan jin teng decoction san alongside Tibetan medicine Xiao bi ointment improved overall clinical outcomes by relieving joint pain and enhancing joint functionality.

#### 6.2.2 Miao medicine, Mongolian medicines and Dong medicines

Wu Teng ointment, a traditional Miao medicine (Zhou J. et al., 2017), was investigated by Gu et al. (2017) for its clinical efficacy in combination with specific electromagnetic wave therapeutic apparatus (TDP) for the treatment of RA. Based on a table of randomly selected numbers, 80 RA patients were divided into two groups: the therapy group and the vehicle control group. The clinical effectiveness of the two groups was assessed after 2 weeks. The findings demonstrated that the combined use of Wu Teng ointment and TDP effectively improved joint inflammation and enhanced clinical outcomes in RA patients.

Additionally, Narengei (2019) conducted a study involving 120 RA patients admitted between May 2014 and December 2016 to investigate the therapeutic effects of Mongolian medicines (Chao and Bai, 2023; He et al., 2021a) on RA. The results revealed significant clinical efficacy of Mongolian medicines in treating RA, with fewer adverse reactions and a higher safety profile, leading to high recognition among patients regarding their effectiveness.

Yang and Yang (2009) applied Dong nationality medicine (Luo et al., 2023; Zhang, 2011) combined with traditional Chinese medicine to treat RA, and the clinical results show that Dong medicine combined with traditional Chinese medicine in the treatment of RA could control the progression of the disease, and it was an effective method for the treatment of RA.

#### 6.2.3 Wa medicine, Zhuang medicine and Yao medicine

In their application of Wa nationality elimination of toxicant and elimination of RA decoction (Zhao and Ni, 2014; Pang et al., 2010; Guan et al., 2004; Gao et al., 2021; Leng, 2021; Qiu and Tang, 2010; Tang et al., 2018a) in clinical practice, Tang et al. (2018b) discovered that the modified prescription was more clinically effective in reducing clinical symptoms such as morning stiffness time, joint swelling, and joint pain in patients with RA.

To evaluate the effectiveness of applying the Zhuang medicine Long zuan tong bi formula (Luo et al., 2019; Zhang et al., 2020; Li et al., 2024) for the treatment of RA, Pang et al. (2013) randomly separated 80 instances of patients with RA into two groups. They also assessed the clinical symptom scores and efficacy of all patients before and after treatment. The results showed that the prescription had better clinical efficacy in the treatment of RA.

According to Wang et al. (2019a), 80 instances of RA patients who met the observation criteria between June 2016 and December 2017 were split into two groups at random: an observation group and a control group. The control group received methotrexate treatment, while the observation group was given Yao medicine Qian jin bo oil needle (Wang et al., 2019b; Lin, 2022) treatment. It was found that the clinical efficacy of Yao medicine Qian jin bo oil needle in the treatment of RA was better than that of traditional Chinese medicine, which could significantly downregulate the levels of ESR, CRP, RF, and related serum inflammatory factors, and alleviate the inflammatory injury of joint synovium.

#### 6.2.4 Dai medicine, Uyghur medicine and Yi medicine

Pan et al. (2018) observed the clinical efficacy of Dai medicine Ya long Meng sha hou prescription in the treatment of 100 cases patients with RA, and found that the effective rate reached 88% after treatment, and the patients’ time of morning stiffness, the number of joint pressure pains, and the number of swollen joints and pains decreased significantly after treatment, and the indexes of blood sedimentation and indexes of C-reactive protein improved significantly compared with the pre-treatment period, and there were no obvious adverse reactions.

From October 2014 to February 2016, Maimaitijjan and Ainiwaer (2017) treated a total of 62 cases with RA patients admitted with mature formulas of different syndromes of Uyghur medicine (Pataer et al., 2010; Atikanmu et al., 2015), and found that the total effective rate of their treatment results was 80.65%, suggesting that the efficacy of Uyghur medicine in the treatment of RA is exact.

The study conducted by Long and Zhou (2011) aimed to investigate the clinical effectiveness of Yi medicine self-made prescription (Long, et al., 2011) in treating RA by randomly assigning 240 patients with RA cases to treatment groups and control groups. The total effective rate of the treatment group reached 88.33%, and the joint swelling, pain, and mobility of the joints all improved significantly, and the indicators of CRP, ESR, and WBC in the blood all decreased after the treatment, and the overall effect was better than that of the control group, which indicated that the Yi medicine self-proposed prescription was a significant effect in the treatment of RA.

#### 6.2.5 Tujia medicine

92 cases of RA patients admitted between March 2004 and September 2005 were divided into a treatment group and a control group at random by Jiang (2007); the treatment group was mainly treated with the Tujia ethnic medicine Qing jiang Rheumatism Medicated wine (Jiang et al., 2000), and the control group was mainly treated with Zheng Qing Feng Tong Ning tablets, and the cases in the two groups were compared and observed in terms of the total therapeutic efficacy, the main symptoms, physical signs, experimental indexes, and the toxic and side effects. The clinical observation showed that the clinical total effective rate of Qing Jiang rheumatism medicine wine in the treatment of RA was 89.55%, which was significantly higher than that of the control group. In addition, the ESR, CRP, RF, IgG, and IgA indexes were significantly decreased in the treatment group, and the improvement effects of the joint pain, morning stiffness, both hands grip strength, and joint swelling were significantly improved, which was better than the control group.

### 6.3 The effect of non-drug therapies on RA

Non-drug therapies for treating RA in various ethnic minorities encompass medicated baths, sand therapy, sleep therapy, moxibustion, mud therapy, wire point moxibustion, medicated cupping therapy, scalding bath therapy, needle pricking therapy, bloodletting, joint nuo-ha-la-hu-shu therapy and others (Chen et al., 2022; Yan et al., 2023; Bao et al., 2022; Liu et al., 2019; Jiang et al., 2023; Zhang Z. J. et al., 2021; Luo et al., 2020; Su, 2016; Chen T. W. et al., 2018; Nan et al., 2019; Zhu et al., 2020; Li L. et al., 2022). By summarizing and comparing the non-drug treatments across different ethnic groups, it is evident these methods exhibit relative similarities with occasional unique therapies specific to certain ethnicities. This paper describes some commonly employed non-drug therapies among ethnic minorities and examines their impact on RA.

#### 6.3.1 Tibetan medicine

Studies have demonstrated that Tibetan medicine bath (Liu et al., 2023; Geng et al., 2021; Yang et al., 2019) exhibits superior therapeutic efficacy in adjuvant arthritis rats, potentially through its modulation of inflammatory cytokines IL-6 and TNF-α (Chen et al., 2009). The integration of Tibetan medicine baths with acupuncture (Xu D. et al., 2018; Yan et al., 2013) for RA treatment can effectively reduce serum levels of MMP-3, IgG, and RF while improving clinical symptoms; moreover, this approach demonstrates enhanced safety (Fu et al., 2022). Fire moxibustion in Tibetan medicine (Wang, 2015; Chen Y. Q. et al., 2018) involves applying moxa columns on acupoints to stimulate nerves transmitting signals to the brain. This technique promotes regulation functions within the cerebral cortex while balancing “wind,” “gallbladder,” and “phlegm,” ultimately leading to improved clinical symptoms among RA patients by effectively alleviating pain symptoms and aiding disease progression control (Wanma, 2018; Gongbao and Zhaxi, 2019).

#### 6.3.2 Mongolian medicine and Dai medicine

Compared to the control group, which followed a conventional approach of disease management involving medication combination and individualized treatment, the combined therapy of Mongolian sand therapy (Bai, 2020; Huangnao, et al., 2013) with internal Mongolian medicine achieved a higher effective rate of 95.24%, indicating significant therapeutic efficacy and shorter treatment duration, thus warranting wider adoption (Du, 2019). The A’ershan mud therapy of Mongolian medicine (Xilintuya et al., 2023) demonstrates efficacy in pain relief, reduction of swelling, antibacterial activity, anti-inflammatory effects, diuretic properties, and wind expulsion. It exhibits a favorable therapeutic effect on the treatment of RA (Bao et al., 2022).

In the case of Dai medicine sleeping therapy (Zhang L. et al., 2021; Tao, 2015) combined with Western medicine for RA treatment, morning stiffness duration as well as joint tenderness, swelling, and pain all significantly decreased compared to pre-treatment levels; furthermore, this therapy exhibited superior effectiveness when compared to the control group. Sleeping therapy not only improves clinical symptoms but also enhances physical signs and laboratory examination indexes related to RA while boosting overall immune function (Yu et al., 2019).

#### 6.3.3 Zhuang medicine and Tujia medication

Significant improvements were observed in the main symptoms and physical signs of RA patients after Zhuang medicine line-point moxibustion (Pan et al., 2021; Xu et al., 2015; Zhong et al., 2021; Xie et al., 2019) treatment, with notable reductions in blood sedimentation rate, C-reactive protein levels, and rheumatoid factor levels. The effective rate reached 96.7%. Furthermore, bamboo canister medication (Chen, 2024; Tang et al., 2021; Huang, 2019) significantly reduced serum immunoglobulin levels with a therapeutic effectiveness rate of 93.33% for RA treatment (Ma et al., 2018).

In comparison to ibuprofen from Western medicine, Tujia nationality Lei Huo Shen Needle (Zhang Y. et al., 2018) demonstrated comparable efficacy in alleviating bi-complex paralytic pain associated with rheumatism while surpassing ibuprofen extended-release tablets in improving pain index, swelling index, pressure index grip index, and site index among patients (Peng et al., 2011).

**FIGURE 4:**
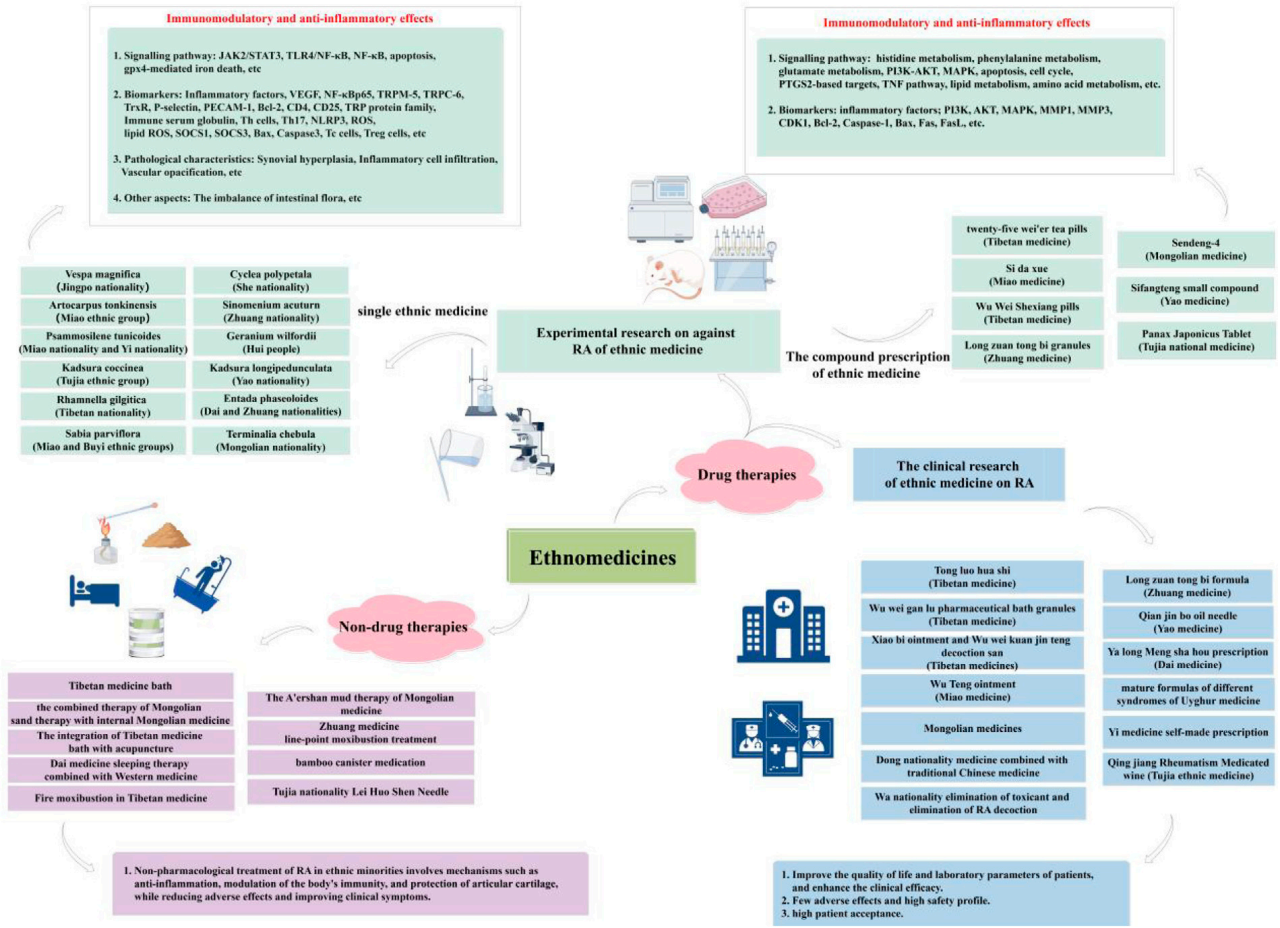
Ethnomedicine-based approaches for RA treatment.

To sum up, non-pharmacological treatment of RA in ethnic minorities involves mechanisms such as anti-inflammation actions, modulation of the body’s immune system, and preservation of articular cartilage, among others. Additionally, it aims to minimize adverse reactions and enhance clinical symptoms, making it a widely employed approach in the clinical management of RA. The aforementioned represents only a fraction of the available non-drug therapies. However, diverse ethnic populations have accumulated extensive experience in this domain with commendable outcomes (Figure 4).

## 7 Summary and prospect

In summary, RA is a chronic, progressive autoimmune disease characterized by aggressive pathology. Its incidence has been steadily increasing over the years. The pathogenesis of RA may involve immune cell dysfunction, inflammatory mediators, matrix metalloproteinases, oxidative stress abnormalities, genetic factors, and environmental influences on survival. However, due to the complex and unclear mechanisms underlying this disease’s pathogenesis, diagnosis and treatment pose significant challenges. Currently, available chemical drugs and biological products primarily target enzymes involved in inflammation regulation as well as inflammatory signaling pathways to mitigate RA symptoms. Nevertheless, these therapeutic agents exhibit certain toxicities and side effects that limit their efficacy in treating RA.

Compared to chemical drugs, TCM and ethnic medicine not only possess abundant resources and exhibit remarkable curative effects, but also offer the advantages of minimal side effects, multi-component formulations, and multi-target actions. They constitute an integral part of complementary and alternative medicine. With increasing attention being paid to ethnic medicine by the country, both domestic and international scholars as well as pharmaceutical companies have shown a strong interest in Chinese herbal medicine, which has emerged as a significant source for innovative drug discovery. This article reviews the impact of ethnic medicine on the treatment of RA. Ethnic medicine along with its non-drug therapies form a comprehensive system for treating RA using medicinal substances. Among these approaches, ethnic medicine shares similarities with TCM in terms of RA treatment strategies, predominantly relying on compound prescription therapy. Tibetan, Mongolian, Miao, Zhuang, and other ethnic medicines already possess relatively complete theoretical systems. Consequently, they hold distinct advantages in documenting and preserving prescriptions and medicinal materials used for treating RA, however, there are comparatively fewer literature studies or records available for other ethnic groups. In terms of therapeutic methods employed by various ethnicities against RA symptoms, most advocate a combination approach involving oral administration alongside external treatments. Furthermore, the research on curative effects and mechanisms underlying the use of ethnic medicine primarily focuses on compound prescriptions rather than individual herbs. Thus, reports regarding single herb usage are scarce while more extensive investigations are lacking, particularly systematic studies exploring their pharmacodynamic material basis or discussing their mechanism of action.

At present, numerous monomer compounds or bioactive components derived from ethnic medicines have been utilized for the treatment of RA. Exploring novel drug candidates from traditional medicines, particularly ethnic folk medicines, represents a crucial approach to innovative drug research and the discovery of new therapeutic agents. Over an extended period of clinical practice and life experience, ethnic medicine has accumulated valuable insights into RA treatment and demonstrated unique efficacy. By employing methods such as syndrome differentiation-based treatment, staged therapy, and comprehensive approaches to manage RA, not only remarkable curative effects are achieved but also toxicities and side effects can be effectively reduced. Consequently, ethnic medicine has gained widespread application in clinical practice. Presently, researchers delve deeper into the utilization of ethnic medicines for RA treatment and discover that investigations on their mechanisms primarily revolve around tumor necrosis factor and interleukin modulation; matrix metalloproteinases regulation; involvement of other related factors including enzymes and inflammatory mediators; signaling pathway modulation; as well as impact on intestinal flora. However, due to the distinct regional characteristics of ethnic areas where independent languages are still predominantly used without systematic development or transmission through writing systems or standardized language forms—ethnic medicine remains incompletely explored and organized to a certain extent—which hampers its inheritance and promotion.

Due to the lack of uniformity and standardization, the research on medicines of various ethnic nationalities lacks systematicity. The quality standard system for various ethnic herbs is inadequate, and there is insufficient uniformity in disease symptoms and names. Clinical research primarily focuses on observing therapeutic effects, which significantly restricts the clinical application of ethnic medicines for treating RA. Furthermore, the unclear identification of the “effective form” of active substances in certain ethnic medicines and their preparations hinders further research, promotion, as well as comprehensive development, and utilization.

Therefore, we propose that future research should focus on enhancing the clinical investigation and case studies of ethnomedicines regarding their curative principles and therapeutic approaches for treating RA. Additionally, it is crucial to strengthen research efforts concerning the material basis of pharmacodynamic effects of ethnomedicines in intervening with RA and elucidating their mechanisms of action. Furthermore, standardizing medication therapy methods and diagnostic criteria is essential to facilitate the clinical application and promote the development of ethnomedicines in treating RA.

## Abbreviations

RA, rheumatoid arthritis; NSAIDs, non-steroidal anti-inflammatory drugs; SAARDs, slow-acting anti-rheumatic drugs; TCM, traditional Chinese medicine; ROS, reactive oxidative stress; DMARDs, disease-modifying antirheumatic drugs; GCs, glucocorticoids; WV, Vespa magnifica; CIA, collagen-induced arthritis; IL-1β, interleukin-1β; TNF-α, tumor necrosis factor-α; IL-8, interleukin-8; IL-6, interleukin-6; COX-2, cyclooxygenase-2; PGE2, prostaglandin E2; ASC, apoptosis-associated speck-like protein; INF-γ, Interferon-γ; VEGF, vascular endothelial growth factor; NF-κB p65, nuclear factor kappa-B p65; TRPM-5, transient receptor potential melastatin-5; TRPC-6, transient receptor potential channel-6; CD62P, P-selectin; PECAM-1, platelet endothelial cell adhesion molecule 1.
