# Supplementary material for: Research overview of ethnic medicines for the treatment of rheumatoid arthritis
Source: Front Pharmacol. 2025 Sep 23;16:1662130. doi: 10.3389/fphar.2025.1662130 (PMC12500662; doi:10.3389/fphar.2025.1662130)
Supplement: Supplementary file 1 [file Table1.docx]

**Supplementary materials For *Research Overview of Ethnic Medicines for the Treatment of Rheumatoid Arthritis***

**Supplementary Table 1.**

The Summary Table of Single herb / Compound prescription for Rheumatoid Arthritis by Ethnic Minorities. (Categorized by ethnic groups)

| **Single herb / Compound prescription (Name)** | | **Composition** | **References** |
| --- | --- | --- | --- |
| **Tibetan Medicine** | | | |
| 1 | Weng bu (Ramulus Myricariae) | *Myricaria germanica* (L.) Desv belongs to the genus *Myricaria* family which is a Part of the dry-ground | Huang. (2009) |
| 2 | Yi shou cao (Hooker Winghead Herb) | *Pterocephalus hookeri* (C.B.Clarke) Hoeck*.* | Shen. (2002) |
| 3 | Hui mao dang sheng (Codonopsis canescens) | *Codonopsis canescens* Nannf*.* | Wang et al. (2022) |
| 4 | Kuan jin teng (Chinese Tinospora Stem) | *Tinospora sinensis* (Lour.) Merr. | Gesang et al. (2017) |
| 5 | Qin jiao (Largeleaf Gentian Root) | [*Gentiana*](https://www.sciencedirect.com/topics/pharmacology-toxicology-and-pharmaceutical-science/gentiana)*macrophylla*Pall*.* | Zhao et al. (2015) |
| 6 | Huang shu kui (sunset abelmoschus) | *Abelmoschus manihot* (L.) Medik*.* | Wang et al. (2022) |
| 7 | Ru yi zhen bao wan | *Concha Margaritifera Usta*, *Aquilaria agallocha* (Lour.) Roxb*,* Travertine*, Lapis Micae Aureum, Carthamus tinctorius* L*., Brachyura, Eugenia caryophyllata* Thunb*., Terminalia billerica* (Gaertn.) Roxb*.* (Go to core), *Myristica fragrans* Houtt., *Amomum kravanh* Pierre ex Gagnep*., Phyllanthus emblica* L*., Amomum tsao-ko* Crevost et Lemaire*, Cuminum cyminum* L*., Santalum album* L*., Nigella glandulifera* Freyn et Sint*., Dalbergia odorifera* T.Chen.*, Terminalia chebula* Retz*., Alpinia officinarum* Hance*., Glycyrrhiza uralensis* Fisch. paste*, Cinnamomum cassia* Presl.*, Boswellia carterii* Birdw*., Aucklandia lappa* Decne*., Cassia obtusifolia* L*., Bubalus bubalis* Linnaeus.*, Abelmoschus manihot* (Linn.) Medicus.*, Lagotis brachystachya* Maxim*., Inula racemosa* Hook.f*., Moschus berezovskii* Flerov (artificial).*, Bos taurus domesticus* Gmehin. | Lu and Zhang. (2010) |
| 8 | Wu wei le zhe tang san | *Tinospora cordifolia* Miers*., Terminalia chebula* Retz*., Swertia mussotii* Franch*., Terminalia bellirica* (Gaertn.) Roxb*., Phyllanthus emblica* L*.,* 5 g each. | Xiao-Wu. (2017) |
| 9 | Chinese and Tibetan medicine Shi wei meng peng powder | 4 copies of *Terminalia chebula* Retz*.*, 4 copies of *Aconitum kusnezoffii* Reichb., 1 copy of *Rhamnella gilgitica* Mansf. et Molch.*,* 0.5 copies of *Moschus berezovskii* Flerov*.,* 1 copy of *Styrax tonkinensis* (Pierre) Craib ex Hart*.,* 2 copies of *Acorus calamus* L*.,* 1 copy of *Boswellia carterii* Birdw*.,* 1 copy of *Aucklandia lappa* Decne*.,* 1 copy of *Cassia obtusifolia* L*.,* 1 copy of *Abelmoschus manihot* (Linn.) Medicus. | Ma. (2001) |
| 10 | Wu wei Gan Lu Liquid | *Rhododendron simsii* Planch*., Myricaria germanica, Sabina przewalskii* Kom*., Ephedra sinica* Stapf*, and Artemisia capillaris* Thunb*.,* which are fermented in distiller's yeast, and packed in sand bags. Put it into a steam pot with water of about 500 L, soak it for 30 min, then put it into high-pressure steam and boil it for 1h. Pour the boiled liquid into the bath, take another 500 ml of grain white wine, and add *Moschus berezovskii* Flerov. 0.5-1 g and soak it for 12 h to make *Moschus berezovskii* Flerov. wine. | Ma. (2009) |
| 11 | Combined internal and external bathing method | **(Internal medicine)** **Si wei Tibetan mu xiang decoction:** *Inula racemose* Hook. f. 100 g, *Rubus kokoricus* Hao*.* 30 g, *Tinospora sinensis* (Lour.) Merr*.* 100 g, *Kaempferia galanga* L*.* 25 g  **(Internal medicine) Er shi wu wei wen guan mu pills:** *Xanthoceras sorbifolia* Bunge (*Acacia catechu* (L.f.) Willci*.*) 100 g, *Terminalia chebula* Retz*.* 100 g, *Terminalia bellirica* (Gaertn.) Roxb*.* 125 g, *Phyllanthus emblica* L*.* 100 g, *Sphallerocarpus gracilis* 50 g, *Polygonatum sibiricum* Red*.* 40 g, *Asparagus cochinchinensis* (Lour.) Merr*.* 40 g, *Oxybaphus himalaicus* Edgew. 25 g, *Tribulus terrestris* L．30 g, *Melicope pteleifolia* 50 g, *Cassia obtusifolia* L*.* 50 g, *Abelmoschus Manihot* (L.) Medic. 35 g, *Tinospora sinensis* (Lour.) Merr*.*100 g, *Piper longum* L*.* 30 g, iron dust (made) 15 g, *Ochotona erythrotis* Btichner. 50 g, *Aconitum szechenyianum* Gay*.* 40 g, *Moschus berezovskii* Flerov. 1 g, *Acorus calamus* L*.* 50 g, *Saussurea costus* (Falc.) Lipech*.* 50 g, *Hyriopsis cumingii* (Lea) 25 g, *Oxytropis kansuensis* Bunge. 40 g, *Rosa sweginzowii* Koehne. 50 g, *Gentiana straminea* Maxim*.* 30 g  **(Internal medicine) Er shi wei lv xue pills:** *Equus asinus* Linnaeus (2-3 years old) blood 25 g (dry), *Santalum album* L*.* 25 g, *Pterocarpus indicus* Willd*.* 40 g, *Terminalia chebula* Retz*.*75 g, *Terminalia bellirica* (Gaertn.) Roxb*.* 50 g, *Phyllanthus emblica* L*.* 40 g, *Bambusa textilis* McClure. 50 g, *Eugenia caryophyllata* Thunb*.* 15 g, *Myristica fragrans* Houtt*.* 15 g，*Amomum kravanh* Pierre ex Gagnep*.* 15 g，*Amomum tsao-ko* Crevost et Lemaire. 15 g，*Melicope pteleifolia* 25 g，*Cassia obtusifolia* L*.* 25 g，*Abelmoschus manihot* (Linn.) Medicus. 25 g，*Gossampianus malabarica* DC. Merr. 15 g, *Gossampinus malobarica* (DC.) Merr. 15 g, *Gentiana manshurica* Kitag. 40 g, *Pterocephalus hookeri* (C.B.Clar ke)Hoeck 35 g, *Fraxinus rhynchophylla* Hance. 40 g, *Saxifraga umbellulate* Hook.f.et Thoms*.* 35 g, *Corydalis impatiens* (pall.) Fisch*.* 35 g, *Tinospora sinensis* (Lour.) Merr*.* 50 g, *Bos taurus domesticus* Gmehin. 1 g, *Moschus berezovskii* Flerov. 1 g, *Crocus sativus* L. 10 g.  **(Internal medicine) Shi wu wei yun peng pills:** *Melicope pteleifolia* (Champ. ex Benth.) Hartley. 35 g, *Cassia obtusifolia* L*.* 35 g, *Abelmoschus Manihot* (L.) Medic*.*35 g, *Saussurea costus* (Falc.) Lipech*.* 5 g, *Corydalis impatiens* (Pall.) Fisch. 30 g, *Terminalia chebula* Retz. 100 g, *Terminalia bellirica* (Gaertn.) Roxb*.* 40 g, *Phyllanthus emblica* L*.* 30 g, *Tinospora sinensis* (Lour.) Merr*.* 10 g, *Ochotona erythrotis* Btichner. 5 g, *Styrax tonkinensis* (Pierre) Craib ex Hart*.* 25 g, *Aconitum szechenyianum* Gay*.* 100 g, *Acorus calamus* L*.* 15 g, *Moschus berezovskii* Flerov. 10 g, *Acacia catechu* (L.f.) Willci. 35 g.  **(External Bathing)** *Rhododendron lutescens* Franch*.* (*Rhododendron anthopogonoides* Maxim*.*), *Myricaria* Desv*.*, *Juniperus chinensis* L*.* (*Juniperus formosana* Hayata), *Ephedra gerardiana* Wall*., Erigeron annuus* (L.) Pers*.,* 500-1000 g of each of the 5 herbs*,* depending on the severity of the disease, adding 500 g of *Xanthoceras sorbifolia* Bunge., after boiling in a high-pressure steamer for 1h, put the liquid into a bath. When the water temperature is adjusted to 38-42 ℃ or when the patient can enter the basin, first pour 25 ml of barley wine, and 500 ml of warm water soaked in 0.5-1 g of *Moschus berezovskii* Flerov. infusion of 25 ml and 10 g of prepared Wu gen san and 10 g of repellent yellow water san into the basin at the same time and stir well. | Wan-Ma. (1999) |
| 12 | Shi wei ru xiang San | *Boswellia carterii* Birdw*.* 100 g, *Terminalia chebula* Retz*.* (denucleation) 150 g, *Cassia obtusifolia* L*.* 80 g, *Terminalia bellirica* (Gaertn.) Roxb*.* 100 g, *Abelmoschus Manihot* (L.) Medic. 80 g, *Phyllanthus emblica* L*.* 120 g, *Aucklandia lappa* Decne*.* 85 g, *Tinospora sinensis* (Lour.) Merr*.* 100 g, *Adhatoda vasica* Nees. 80 g, brag-zhun paste 50 g. The above 10 medicines are crushed into fine powder, sieved, and mixed to obtain. | Dan. (2009) |
| 13 | Sang dang nie e | *Acacia catechu* (L.f.) Willci. 100 g, *Terminalia chebula* Retz*.* 100 g, *Terminalia bellirica* (Gaertn.) Roxb*.* 125 g, *Phyllanthus emblica* L*.* 100 g, *Sphallerocarpus* Besser ex DC. 50 g, *Polygonatum sibiricum* Red*.* 40 g, *Asparagus cochinchinensis* (Lour.) Merr*.* 40 g, *Mirabilis himalaica* (Edgew) Heimerl. 250 g, *Tribulus terrestris* L*.* 30 g, *Evodia lepta* (Spreng.) Merr*.* 50 g, *Cassia obtusifolia* L*.* 50 g, *Abelmoschus manihot* (L.) Medic. 35 g, *Tinospora sinensis* (Lour.) Merr*.* 100 g, *Piper longum* L*.* 30 g, iron dust (Self-made) 15 g, brag-zhun Paste 50 g, *Aconitum szechenyianum* Gay*.* 40 g, *Moschus berezovskii* Flerov. 1 g, *Acorus calamus* L*.* 50 g, *Aucklandia lappa* Decne. 50 g, *Rhinocerotis Asiatici Cornu.* 15 g, *Hyriopsis cumingii* (Lea) 25 g, *Oxytropis kansuensis* Bunge. 40 g, *Rosa sweginzowii* Koehne. 50 g, *Gentiana straminea* Maxim*.* 30 g | Ga. (2004) |
| 14 | Zhen wu su jiao | *Terminalia chebula* Retz*.* (enucleation) 2.5 g, *Crocus sativus* L*.* 10 g, *Amomum kravanh* Pierre ex Gagnep*.* 18.5 g, brag-zhun Paste 10 g, *Swertia mussotii* Franch. 10 g, Canavalia *gladiata* (Jacq.) DC*.* 10 g, Shan shi ling leaf 10 g, *Rubia cordifolia* L*.* 10 g, *Laccifer lacca* Kerr. 10 g, *Juniperus formosana* Hayata. 10 g, Borneol 3 g, *Bambusa textilis* McClure. 10 g, *Eugenia caryophyllata* Thunb. 6.5 g, *Myristica fragrans* Houtt. 6 g, *Amomum tsao-ko* Crevost et Lemaire. 8.5 g, *Aquilaria sinensis* (Lour.) Gilg*.* 5 g, *Santalum album* L*.* 9 g, *Pterocarpus indicus* willd. 6.5 g, *Meconopsis quintuplinervia* Regel*.* 5.5 g, *Gossampinus malabarica* (DC.) Merr. 6 g, *Gossypium herbaceum* L. Petals 6 g, *Aucklandia lappa* Decne. 9 g, *Cuminum cyminum* L. 9 g, *Aristolochia debilis* Sieb. et Zucc. 5 g, *Cinnamomum cassia* Presl*.* 9 g, *Rapana thomasiana* Crosse. 6.5 g, *Dendrobium nobile* Lindl. 6.5 g, *Nardostachys chinensis* Batal. 8 g, *Corallodiscus flabellatus* (Craib.) Burtt. 14 g, *Medicago ruthenica* (L.) Trautv. 5 g. Febrile disease plus *Terminalia chebula* Retz. (denucleation) 9 g, *Terminalia bellirica* (Gaertn.) Roxb*.* (denucleation) 5 g, *Phyllanthus emblica* L*.* (denucleation) 6g; Cold disease plus *Piper longum* L. 9 g, *Piper nigrum* L*.* 25 g, *Zingiber officinale* Rosc. 6 g. | Ga. (2008) |
| 15 | Er shi wu wei Lv xue pill (Zhen cai ni ai ri bu) | *Equus asinus LinnaetIs.* 25 g (2–3-year-old donkey), *Santalum album* L*.* 25 g, *Pterocarpus indicus* willd. 40 g, *Terminalia chebula* Retz. 75 g, *Terminalia bellirica* (Gaertn.) Roxb*.* 50 g, *Phyllanthus emblica* L. 40 g, *Bambusa textilis* McClure. 50 g, *Eugenia caryophyllata* Thunb. 15 g, *Myristica fragrans* Houtt. 15 g, *Amomum kravanh* Pierre ex Gagnep*.*15 g, *Amomum tsao-ko* Crevost et Lemaire. 15 g, *Melicope pteleifolia* (Champ. ex Benth.) Hartley. 25 g, *Cassia obtusifolia* L*.* 25 g, *Abelmoschus Manihot* (L.) Medic. 25 g, *Gossampianus malabarica* (DC). Merr. Calyx 15 g, *Gossampinus malobarica* (DC.) Merr. Filaments 15 g, *Gentiana manshurica* Kitag. 40 g, *Pterocephalus hookeri* (C.B.Clar ke)Hoeck 35 g, *Fraxinus rhynchophylla* Hance. 40 g, *Saxifraga umbellulata* Hook.f.et Thoms*.* 35 g, *Corydalis impatiens* (pall.) Fisch*.* 35 g, *Tinospora sinensis* (Lour.) Merr. 50 g, *Bos taurus domesticus* Gmehin. 15 g, *Moschus berezovskii* Flerov. 1g, *Crocus sativus* L. 10 g. The above 25 herbs, in addition to *Bos taurus domesticus* Gmehin., *Moschus berezovskii* Flerov., *Crocus sativus* L*.* separately grinded fine powder, the rest of the total grinded fine powder, sieved, added *Bos taurus domesticus* Gmehin., *Moschus berezovskii* Flerov., *Crocus sativus* L*.* fine powder, mixed well, added the appropriate amount of water pan pills, dried to obtain. | Ma. (1998);  Jin. (2020) |
| 16 | Wu wei she xiang pills (Wu peng pills) | *Moschus berezovskii* Flerov. 10 g, *Terminalia chebula* Retz*.* (denucleation) 300 g, *Aconitum pendulum* Busch. 300 g, *Aucklandia lappa* Decne*.* 100 g, *Acorus calamus* L*.* 60 g. | He et al. (2022) |
| 17 | Internal medicine and Mai xie | **Internal medicine:**  **(Morning)** **Er shi wu wei da tang pills:** *Hypecoum erectum* L*.*, *Aster tataricus* L. f*.*, *Terminalia chebula* Retz., *Inula racemosa* Hook.f*.*, *Drynaria fortunei* (Kunze) J.Sm*.*, *Coriandrum sativum* L*.*, *Aucklandia lappa* Decne*., Punica granatum* L*., Myricaria germanica., Gentiana urnula* H. Smith*., Terminalia bellirica* (Gaertn.) Roxb*., Carthamus tinctorius* L*., Amomum kravanh* Pierre ex Gagnep*., Meconopsis racemosa* Maxim*., Chaenomeles speciosa* (Sweet) Nakai*., Gentiana straminea* Maxim*., Lagotis brachystachy* Maxim*., Swertia mussotii* Franch*., Semen Herpetospermi Pedunculosi.,* brag-zhun Paste, Pig Blood Powder, *Aconitum naviculare* (Bruhl.) Stapf., *Phyllanthus emblica* L., *Geranium pylzowianum* Maxim., *Corydalis impatiens* (Pall.) Fisch*.*  **(Midday) Er shi wu wei Lv xue Pills:** *Rhamnella gilgitica* Mansf. et Molch., *Santalum album* L., *Terminalia bellirica* (Gaertn.) Roxb., *Fraxinus rhynchophylla* Hance*.,* travertine*, subsect. Rosulares Gornall., Phyllanthus emblica* L*., Eugenia caryophyllata* Thunb*., Terminalia chebula* Retz*., Dalbergia odorifera* T.Chen*.,* Artificial musk*, Amomum kravanh* Pierre ex Gagnep*., Boswellia carterii* Birdw*., Gossampinus malabarica* (DC.) Merr*., Crocus sativus* L*., Gentiana scabra* Bunge*., Adhatoda vasica* Nees*., Myristica fragrans* Houtt*., Amomum tsao-ko* Crevost et Lemaire*., Abelmoschus manihot* (L.) Medic*., Tinospora sinensis* (Lour.) Merr*., Cassia obtusifolia* L*., Pterocephalus hookeri* (C.B.Clarke) Hoeck*.,* donkey blood*,* Artificial bezoar.  **(Evening)** **San guo decoction powder:** *Phyllanthus emblica L.*, *Terminalia chebula* Retz., *Terminalia bellirica* (Gaertn.) Roxb*.*  **Ba wei xue wa Pills:** *Canavalia ensiformis., Carapax Eriocheir sinensis.,* Broken mica in the stream, *Malva verticillata* L*., Elettaria cardamomum* (L.) Maton*., Syzygium jambos* (L.) Alston*., Chinese Forest Frog.,* Zha ku xiu*,* Sugar is lead. (Patients with kidney disease and distorted, obstructed or knotted urinary tract can be taken together.)  **(Medicinal bath)** **Wu wei Gan lu decoction:** *Ephedra sinica* Stapf*.*, *Juniperus formosana* Hayata., *Artemisia sieversiana* Ehrhart ex Willd., *Rhododendron anthopogonoides* Maxim*.*, *Myricaria germanica* (L.) Desv*.*  Treatment of Mai xie in Tibetan medicine. | Chen et al. (2021) |
| 18 | Er shi wu wei er cha Pills | stem of *Senegalia catechu* (L.f.) P.J.H. Hurter and Mabb*.* (100 g), fruits of *Terminalia chebula* Retz*.* (100 g), fruits of *Terminalia Billerica* (Gaertn.) Roxb*.* (125 g), fruits of *Phyllanthus emblica* L*.* (100 g), *Hymenidium hookeri* (C.B.Clarke) Pimenov and Kljuykov*.* (50 g), rhizome of *Polygonatum sibiricum Redouté* (40 g), root of *Asparagus cochinchinensis* (Lour.) Merr*.* (40 g), root of *Oxybaphus himalaicus* Edgew. (25 g), fruits of *Tribulus terrestris* L*.* (30 g), resin of *Boswellia sacra Flück. (Boswellia carterii* Birdw*.)* (50 g), seeds of *Senna tora* (L.) Roxb*.* (50 g), seeds of *Abelmoschus manihot* (L.). Medik*.* (35 g), stem of *Tinospora sinensis* (Lour.) Merr*.* (100 g), fruits cluster of *Piper longum* L*.* (30 g), the rhizome of *Acorus calamus* L*.* (acori calami rhizoma) (50 g), root of *Dolomiaea costus* (Falc.) Kasana and A.K.Pandey (aucklandiae radix) (50 g), *Oxytropis kansuensis* Bunge. (40 g), fruits of *Rosa sweginzowii* Hemsl. and E.H.Wilson. (50 g), flower of *Gentiana macrophylla* Pall*.* (30g), the root of *Aconitum pendulum* N.Busch (*Aconitum pendulum* Busch.) (40 g), the musk of *Moschus chrysogaster sifanicus.* (30 g), the shell of *Hyriopsis cumingii* (Lea) (25 g), and the horn of *Bubalus arnee* f. bubalis. (25 g). | Li et al. (2022) |
| 19 | Compound Ru Teng (CRT) | *Boswellia carterii* Birdw*., Tinospora sinensis* (Lour.) Merr*., Cassia obtusifolia* L*.,* Abelmoschus manihot (L.) Medic*., Terminalia* chebula Retz*.,* Lamiophlomis rotate (Benth.) Kudo. and Pyrethrum tatsienense (Bur. et Franch.) Ling*..* | Huang et al. (2021) |
| 20 | Tong Luo Hua Shi (TLHS) | Salvia miltiorrhiza., Ephedra intermedia., Sabina przewalskii., Myricaria paniculata., Artemisia sieversiana., Astragalus membranaceus., and Rhododendron anthopogonoides. | Liu et al. (2016) |
| 21 | Wu Wei Gan Lu (medicinal bath) | Ajania tenuifolia (Jacq.) Tzvel., Myricaria germanica (L.) Desv., Ephedra saxatilis Royle ex Florin., Juniperus formosana Hayata., Rhododendron capitatum Maxim., fermentation of the above drugs with distiller’s yeast, 3 days later, then take 1 kg each into the gauze bag, put it into a steam pot, soak for about 40 minutes, and cook for 1 hour with high-pressure steam pot. | Xian. (2020) |
| 22 | Tibetan medicine external application prescription | Juniperus formosana Hayata., Rhododendron simsii Planc h., Arteimiaisia sieuersiana Ehrhart ex Willd., Ephedra saxatilis Royle ex Florin., Myricaria germanica (L.) Desv. The above medicinal materials were stir-fried and heated to 35 ℃ to 43 ℃, and then put into a bag for hot compress on the patient's limbs and affected areas. | Yangben. (2016) |
| [**Mongolian medicine**](javascript:%20void(0)) | | | |
| 1 | He zi (Medicine Terminalia Fruit) | Terminalia chebula Retz. | Liu et al. (2020) |
| 2 | Qin jiao (Largeleaf Gentian Root) | the water extracts from roots of *Gentiana macrophylla* Pall*.* | Zhao et al. (2015) |
| 3 | Zhong lun wu decoction | *Sophora flavescens* Ait., *Gardenia jasminoides* J. Ellis., *Terminalia chebula* Retz., *MeLia toosendan* Sieb.et Zucc., *Swertia pseudochinensis* Hara. | Liu. (2011) |
| 4 | Hua jiao-6 | *Sophora flavescens* Ait., *Gardenia jasminoides* J. Ellis., *Terminalia chebula* Retz., *MeLia toosendan* Sieb.et Zucc., *Gentiana macrophylla* Pall., *Carthamus tinctorius* L. |  |
| 5 | Bie chong zhao na | *Sophora flavescens* Ait., *Terminalia chebula* Retz., *MeLia toosendan* Sieb.et Zucc., *Gardenia jasminoides* J. Ellis., *Aucklandia lappa* Decne., *Faecas Trogopterori*., *Ruta graveolens* L*..* | Qi. (2014) |
| 6 | Compound Sendeng-4 soup | *Xanthoceras sorbifolia* Bunge. (Mongolia named: Xi La **·** Sen deng)., *Gardenia jasminoides* J. Ellis., *Terminalia chebula* Retz., *MeLia toosendan* Sieb.et Zucc. | Bai et al. (2015) |
| 7 | Mongolian medicine Tuolei-15 | *Aconitum kusnezoffii* Reichb., *Terminalia chebula* Retz., *Sarcandra glabra* (Thunb.) Nakai., *Aquilaria sinensis* (Lour.) Gilg., Magnetite, *Sophorae Flavescentis* Radix. and other 15 kinds, warm water to take, developed powder through 100-200 mesh sieve, made of soybean size water pills for later use. | Qi et al. (2003) |
| 8 | Bang ri ga-25 wei; E Er dun wu ri le-29 wei; Wu li chu-18 wei | “Bang ri ga-25 wei” takes donkey blood (dry) as the main prescription; “E Er dun wu ri le-29 wei” contains *Abutilon theophrasti* Medic., *Liquidambar formosana* Hance., *Cassia obtusifolia* L., pearl, *Moschus berezovskii* Flerov., *Aquilaria sinensis* (Lour.) Gilg., *Crocus sativus* L., etc. “Wu li chu-18 wei” contains mercury, sulfur, *Aconitum kusnezoffii* Reichb., black and white *Ruta graveolens* L., *Crocus sativus* L., *Glycyrrhiza uralensis* Fisch., etc. | Bao. (2003) |
| 9 | Mongolian Medicine 25 Wei Lv xue Pills | The main drug is Mongolian medicine 25 Wei Lv xue Pills, dialectical addition and subtraction oral. Ruo he yi and Ba da gan are prevalent, add *Saposhnikovia divaricate* (Turcz.) Schischk. 10 g, *Notopterygium incisum* Ting ex H.T.Chang*.* 10 g, *Aconitum kusnezoffii* Reichb*.* 10 g, Ge ci 10 g; Xi la is prevalent, and *Bubalus bubalis* Linnaeus 10 g, *Anemarrhena asphodeloides* Bge. 10 g; qi and blood deficiency plus *Kadsurae caulisk* 10 g, *Astragalus membranaceus* (Fisch) Bge*.* var*. Mongholicus* (Bge) Hsiao*.,* 10 g; for those with poor joint activity, 10 g of *Lycopodium japonicum* Thunb. and 10 g of *Speranskia tuberculata* （Bunge）Baill. were added; severe pain plus *Boswellia carterii* Birdw. 10 g, *Commiphora myrrha* Engl*.* 10 g; stubborn refractory adds *Pteris vittata* L. 10 strips, *Buthus martensii* Karsch. 5 g. | Wu. (2010) |
| 10 | Beng · ri a ge-25 | *Santalum album* L. 15 g, *Pterocarpus indicus* willd. 15 g., *Gypsum Fibrosum* 10 g., *Crocus sativus* L. 15 g., *Eugenia caryophyllata* Thunb. 10 g, *Myristica fragrans* Houtt. 10 g, *Amomum tsao-ko* Crevost et Lemaire. 10 g., *Liquidambar formosana* Hance. 15 g, *Cassia obtusifolia* L. 15 g, *Abutilon theophrasti* Medic. 15 g, *Terminalia chebula* Retz. 10 g, *Gardenia jasminoides* J*.* Ellis*.* 10 g, *MeLia toosendan* Sieb.et Zucc. 10 g, *Citrus reticulata* Blanco. 10 g, *Magnolia biondii* Pamp. 10 g, *Sophorae Flavescentis* Radix. 10 g, *Hyoscyamus niger* L. 10 g, *Althaea rosea* (L.) Cav. 10 g, *Hosta plantaginea* (Lam.) Aschers. 10 g, *Eucommia ulmoides* Oliv. 10 g, *Corydalis bungeana* Turcz. 10 g, *Amomum kravanh* Pierre ex Gagnep. 10 g, *Bos taurus domesticus* Gmehin. 3 g, *Moschus berezovskii* Flerov*.* 10 g, Dry donkey blood 25 g. The medicine except *Moschus berezovskii* Flerov. and *Bos taurus domesticus* Gmehin. together to research fine surfaces, then blend into the *Moschus berezovskii* Flerov., *Bos taurus domesticus* Gmehin. stirred into a water pill bean grain size. | Hai and Dai. (1996) |
| 11 | Combined use of prescriptions | **A ga ri-35 wei:** 35 kinds of medicines, including *Aquilaria sinensis* (Lour.) Gilg. 12 g, *Santalum album* L. 12 g, *Carthamus tinctorius* L. 2 g, *Aconitum kusnezoffii* Reichb. 2 g, *Moschus berezovskii* Flerov. 0.9 g, etc.  **Lv xue gan-25 wei:** 25 kinds of medicines, including Donkey blood dry 15 g, *Crocus sativus* L. 15 g, *Eucommia ulmoides* Oliv*.* 12 g, etc.  **Zhen zhu tong luo pills:** 29 kinds of medicines, including  *Concha Margaritifera* 2 g, *Gardenia jasminoides* J. Ellis. 2 g, *Cinnamomum cassia* Presl. 6 g, *Aristolochia debilis* Sieb. et Zucc. 6 g, *Lygodium japonicum* (Thunb.) Sw. 6 g, etc.  **Wu li chu-18 wei:** 18 kinds of medicines, including mercury (made) 6 g, *Acorus calamus* L. 12 g, gypsum 6 g, *Eugenia caryophyllata* Thunb. 6 g, etc. | Wange. (2007) |
| 12 | Ben Cao Zha Chong -13 pills combined with Mongolian medicine bath | *Aconitum kusnezoffii* Reichb., *Terminalia chebula* Retz., *Acorus tatarinowii* Schott., *Aucklandia lappa* Decne., *Moschus berezovskii* Flerov., etc. Mongolian medicine bath consists of *Hex chinensis* Sims*.*, *Juniperus formosana* Hayata., *Myricaria paniculata* P.Y.Zhang et Y.J.Zhang., *Ephedra sinica* Stapf. and *Artemisia frigida* Willd. | Qi. (2011) |
| 13 | Rheumatism I capsule plus bath therapy | (prescription) *Clematis chinensis* Osbeck., *Chaenomeles speciosa* (Sweet) Nakai., *Paeonia lactiflora* Pall.; *Aconitum carmichaeli* Debx., *Notopterygium incisum* Ting ex H.T.Chang., *Angelica pubescens* Maxim.f. *biserrata* Shan et Yuan., *Corydalis yanhusuo* W.T. Wang., *Angelica sinensis* (Oliv.) Diels., *Aconitum carmichaelii* Debx.; *Astragalus membranaceus* (Fisch.) Bge*.* var. *Mongholicus* (Bge.) Hsiao., *Spatholobus suberectus* Dunn., *Glycyrrhiza uralensis* Fisch., *Saposhnikovia divaricate* (Turcz.) Schischk., *Ephedra sinica* Stapf., *Atractylodes lancea* (Thunb.) DC., *Coix lacryma-jobi* L.var*.mayuen* (Roman.) Stapf., *Cinnamomum cassia* Presl., *Cyathula officinalis* Kuan., *Acanthopanax gracilistylus* W.W.Smith., *Eucommia ulmoides* Oliv., *Salvia miltiorrhiza* Bge., *Bungarus multicinctus* Blyth., *Zaocys dhumnades* (Cantor)., etc. are made into capsules at a ratio of 3:2:4:1.  (Medicated bath) *Ligustrum lucidum* Ait. 200 g, *Juniperus formosana* Hayata. 200 g, *Cacumen Tamaricis*. 400 g, *Ephedra sinica* Stapf. 400 g, *Artemisia argyi* Levl.et Vant. 400 g, 200 g of sulfur, 200 g of donkey bone, 200 g of *Evodia lepta* (Spreng.) Merr., 200 g of *Cassia obtusifolia* L., and 200 g of *Abutilon theophrasti* Medic*.* and 200 g of *Rutagraveolens* L. were prepared. | Dai. (2007) |
| 14 | Wu wei gan lu (medicinal bath) | *Rhododendron lutescens* Franch., *Myricaria germanica* (L.) Desv., *Juniperus chinensis* Roxb., *Ephedra sinica* Stapf., *Artemisia leucophylla* (Turcz. ex Bess.) C.B. Clarke., 0.8-1 kg each time; in addition, other formulations were added according to the syndrome differentiation of the disease: *Glycyrrhiza uralensis* Fisch. 15 g, *Angelica pubescens* Maxim.f. *biserrata* Shan et Yuan. 12 g, *Smilar glabra* Roxb. 20 g, *Rehmannia glutinosa* Libosch. 10 g, *Scrophularia ningpoensis* Hemsl. 10 g, *Eugenia caryophyllata* Thunb. 10 g, *Zanthoxylum bungeanum* Maxim. 10 g, *Angelica sinensis* (Oliv.) Diels. 10 g, *Salvia miltiorrhiza* Bunge. 20 g, *Artemisia argyi* Levl.et Vant. 30 g, *Lycopodium japonicum* Thunb. 30 g, *Dictamnus dasycarpus* Turcz. 15 g, *Cassia obtusifolia* L. 20 g, etc. | Naren. (2019) |
| 15 | Yi he ha ri-12 | *Sus scrofa* L., *Inula helenium* L., *Corydalis bungeana* Turcz., *Picrorhiza scrophulariiflora Pennell*., *Terminalia chebula* Retz., *MeLia toosendan* Sieb. et Zucc., *Gardenia jasminoides* J. Ellis., Artificial bezoar, Cow gall powder, plaster, *Carthamus tinctorius* L., *Nardostachys jatamansi* DC. | Na et al. (2020) |
| 16 | Chen xiang an shen powder | *Aquilariasinensis* (Lour.) Spreng., *Pterocarpus indicus* willd., *Carthamus tinctorius* L., *Amomum kravanh* Pierre ex Gagnep., *Terminalia chebula* Retz., *Inula japonica* Thunb., *Asarum sieboldii* Miq., *Aconitum kusnezoffii* Reichb., *Gossampinus malabarica* (DC.) Merr., *Picrorhiza scrophulariiflora Pennell*., *Resin Commiphorae muakulis* (Guggulum)., *Liquidambar formosana* Hance., *Syringa pinnatifolia* Hemsl., *Santalum album* L., Plaster, *Myristica fragrans* Houtt., *Amomum tsao-ko* Crevost et Lemaire., *Gardenia jasminoides* J*.* Ellis., *Pulsatilla chinensis* (Bge.) Regel., *Dianthus superbus* L., *Punica granatum* L., *Glehnia littoralis* Fr. Schmidt ex Miq., *Eugenia caryophyllata* Thunb., *Aucklandia lappa* Decne., *Viola yedoensis* Makino., *Sophorae Flavescentis* Radix., *MeLia toosendan* Sieb.et Zucc., *Rubus sachalinensis* Leveille., *Kaempferia galanga* L., *Choerospondias axillaris* (Roxb.) Burtt et Hill., Rabbit heart, *Inula helenium* L., *Moschus berezovskii* Flerov*.*, *Dalbergia odorifera* T.Chen., *Strychnos nux-vomica* L. |  |
| 17 | Cha gan gu gu le-10 | *Liquidambar formosana* Hance., *Cassia obtusifolia* L., *MeLia toosendan* Sieb.et Zucc., *Abutilon theophrasti* Medic., *Aucklandia lappa* Decne., *Sophorae Flavescentis* Radix., *Terminalia chebula* Retz., *Gardenia jasminoides* J. Ellis., *Dianthus superbus* L., *Faecas Trogopterori*. |  |
| 18 | Qi su-25 | Donkey blood powder, *Santalum album* L., *Pterocarpus indicus* willd., *Sophorae Flavescentis* Radix., *Gardenia jasminoides* J*.* Ellis., *Rhododendron molle* G.Don., *Bos taurus domesticus* Gmehin., *Crocus sativus* L., *Amomum tsao-ko* Crevost et Lemaire., *Amomum kravanh* Pierre ex Gagnep., *Viola yedoensis* Makino., *Terminalia chebula* Retz., *MeLia toosendan* Sieb.et Zucc., *Moschus berezovskii* Flerov., *Rhaponticum uniflorum* (L.) DC., *Liquidambar formosana* Hance., *Cassia obtusifolia* L., *Bombax ceiba* Linn., *Eugenia caryophyllata* Thunb., *Eucommia ulmoides* Oliv. |  |
| 19 | Zhuang lun-5 soup | *Sophorae Flavescentis* Radix., *Terminalia chebula* Retz., *MeLia toosendan* Sieb.et Zucc., *Gardenia jasminoides* J. Ellis., *Lomatogonium rotatum* (L.) Fr. ex Nyman. |  |
| 20 | Mongolian medicine bath prescription | *Ilex chinensis* Sims. 750 g, *Platycladus orientalis* (L.) Franco. 750 g, *Myricaria germanica* (L.) Desv*.* 1500 g, *Ephedra sinica* Stapf. 1500 g, *Artemisia frigida* Willd. 2250 g, 100-500 g of each of *Liquidambar formosana* Hance., *Cassia obtusifolia* L., *Abutilon theophrasti* Medic., *Resin Commiphorae muakulis* (Guggulum). and *Xanthoceras sorbifolia* Bunge. | Naren. (2011) |
| 21 | Five-flower medicinal bath | *IIex chinensis* Sims., *Juniperus formosana* Hayata., *Ephedra sinica* Stapf., *Myricaria germanica* (L.) Desv., *Artemisia frigida* Willd. | Xian and Buren. (2006) |
| **Hui medicine** | | | |
| 1 | Chuan shan long (Nippon Yam Rhizome) | *Dioscorea nipponica* Makino. | Ma and Chen. (2005) |
| 2 | Zu shi ma (Bark of Girald Daphne) | *Daphne giraldii* Nitsche. |  |
| 3 | Fan mu bie (Nux Vomica) | *Strychnos nux-vomica* L. | Shan. (2005) |
| 4 | Fu niu hua (Damnacanthus indicus) | The flowers of *Damnacun-thus indicus* Gaertn.f |  |
| 5 | Xun ma (Herba Urticae) | *Urtica fissa* E. Pritz*.* |  |
| 6 | Hai hua (Eucheuma gelatinae) | *Eucheuma muricatum* (Gmel.) Web.van Bos. |  |
| 7 | Cao wu tou (Kusnezoff Monkshood Root) | *Aconitum kusnezoffii* Reichb. |  |
| 8 | Bai fu zi (Giant Typhonium Rhizome) | *Typhonium giganteum* Engl. |  |
| 9 | Chen si ji (Japanese stephania root or leaf) | *Stephania japonica* (Thunb.) Miers. |  |
| 10 | Chai zi gu (morse luisia root or herb) | *Luisia morsei Rolfe Forbes et* Hemsl*.* |  |
| 11 | Hai tong pi  (Oriental Variegated Coralbean Bark) | *Erythrina variegata* L. |  |
| 12 | Lao guan cao ba bu ji (Geranium cataplasm) | *Geranium wilfordii* Maxim. | Yang et al. (2022) |
| 13 | Niu xin piao zi (cynanchum komarovii) | *Cynanchum komarovii* Al.Iljinski. | Zhou et al. (2021) |
| 14 | Can er zi (siberian cocklebur fruit) | *Xanthium sibiricum* Patr., *Morus alba* L., *Geranium wilfordii* Maxim., *Hibiscus trionum* Linn. | Ma and Chen. (2005) |
| 15 | Lao guan cao | *Geranium wilfordii* Maxim., *Phryma leptostachya* L*.* subsp*. asiatica* (Hara) Kitamura., *Angelica pubescens* Maxim.f. *biserrata* Shan et Yuan., *Radix et Rhizoma Clematidis*., *Saposhnikovia divaricate* (Turcz.) Schischk., *Dature Stramonium Datura* L., *Aconitum kusnezoffii* Reichb. |  |
| 16 | Mu jin ji er | *Geranium wilfordii* Maxim., *Morus alba* L., *Tamarix chinensis* Lour. |  |
| 17 | Ye xi gua miao | *Hibiscus trionum* L., *Morus alba* L., *Geranium wilfordii* Maxim. |  |
| 18 | Qie gen | *Solanum melongena* L., *Geranium wilfordii* Maxim. |  |
| 19 | Tie bang chui | *Aconitum szechenyianum* Gay., *Panax notoginseng*（Burk.）F.H.Chen. |  |
| 20 | Lao gua tou | *Cynanchum komarovii* Al. Iljinski., *Geranium wilfordii* Maxim., *Siegesbeckia orientalis* L., *Solanum melongena* L. |  |
| 21 | Feng xian tou gu cao | *Caulis Impatientis Balsaminae*., *Clematis chinensis* Osbeck., *Morus alba* L. |  |
| 22 | Hui xiang | *Foeniculum vuLgare* Mill. (Fennel oil) | Shan. (2005) |
| 23 | Luo tuo peng zi | *Peganum harmala* L. (Oil of *Peganum harmala* L.) |  |
| 24 | Man tuo luo | *Datura metel* L.D.innoxia Mill., *Anisodus acutangulus* C.Y. Wu et C.Chen. |  |
| 25 | Ai na xiang | *Blumea balsamifera* (L) DC., *Blumea balsamifera* DC., *Ricinus commusnis* L., *Acorus tatarinowii* Schott. |  |
| 26 | Yao xiang mao | *Cymbopogon nardus* (L.) Rendle. 500 g. |  |
| 27 | Xue lian hua | *Echeveria laui*. 15 g, White wine 100 ml soak 7 d. |  |
| 28 | Niu xi | *Achyranthes bidentata* Blume., *cinnamomum japonicum* sieb., *Cornus officinalis* Sieb. Et Zucc*.* |  |
| 29 | Shui long gu | *Polypodiodes nipponica* (Mett.) Ching. |  |
| 30 | Jie gu mu | *Sambucus williamsii* Hance *S.williansii* Hance var. (Nakai) Y.C. Tang S.nigra L. 150 g, Fresh tofu 250 g. |  |
| 31 | Zhu ling zhang wan (Qiu shui xian wan) | *Elaeagnus pungens* Thunb., *Bupleurum chinensis* DC., *Colchicum autumnale* L. | Niu. (2010) |
| 32 | Mai ning wan | *Lagenaria sphaerica*., *Ephedra sinica* Stapf., *Cucumis sativus*L., Hei ze mi gang, *Euphorbia pekinensis* Rupr., *Piper nigrum* L., *Ferula teterrima* Kar. et Kir., *Ferula gallbaniflua* Boisset.Buhse., *Corydalis yanhusuo* W.T.Wang., *Semen sinapis*. |  |
| 33 | Shi da ci zhi wan (Yan hu suo wan) | *Bupleurum chinensis* DC., *Terminalia chebula* Retz*.*var*.tomentella* Kurt., *Aloe vera* var*. chinensis* (Haw.) Berg., *Corydalis yanhusuo* W.T.Wang., *Acorus calamus* L., *Lagenaria sphaerica*., *Fomitopsis officinalis* (Vill.:Fr.) Bond., *Ferula teterrima* Kar. et Kir., *Zingiber officinale* Rosc., *Piper nigrum* L., *Piper longum* Linn., *Sinapisalba* L., *Cymbopogon distans* (Nees ex Steud.) Will. Watson., *Silene adenantha* Franch., granulated sugar, Salt. |  |
| 34 | Zha li nu si gao | Watermelon Oil, *Fomitopsis officinalis* (Vill.: Fr.) Bond., *Ornithogalum caudatum* Jacq., *Boswellia carterii* Birdw., *Silene adenantha* Franch., *Veratrum nigrum* Linn., *Malva parviflora* L., *Cuscuta chinensis* Lam., *Teucrium japonicum* Willd*., Spikeflower Germander*., *Lagopsis supina* (Steph.) Ikonn.-Gal. Ex Knorr., *Paeonia suffruticosa* Andr., *Commiphora myrrha* Engl., *Ferula sinkiangensis* K.M.Shen., *Aristolochia debilis* Sieb.et Zucc., *Piper nigrum* L., *Cortex Cinnamomi*., *Ferula gallbaniflua* Boisset. Buhse., Ursine Seal's Penis and Teste, *Angelica sinensis* (Oliv.) Diels., *Aloe vera* var*. chinensis* (Haw.) Berg., *Crocus sativus* L. |  |
| 35 | Wu gen jian (Ma wu li niao su li fang) | *Apium graveolens* L., *Illicium verum* Hook.f., *Cymbopogon citratus* (DC.) Stapf., *Foeniculum vuLgare* Mill., *Apium grauens* L. var. *dulcedc*., *Pistacia chinensis* Bunge., *Nardostachys chinensis* Batal., *Sambucus williamsii* Hance., *Vitis vinifera* L. |  |
| **Zhuang medicine** | | | |
| 1 | Ke teng / Guo gang long (climbing entada stem) | *Entada phaseoloides* (Linn.) Merr. | Xu. (2021) |
| 2 | Qing feng teng (Orientvine Vine) | *Sinomenium acuturn* (Thunb.) Rehd.et Wils. | Cao et al. (2022) |
| 3 | Internal preparation | *Polygonum cuspidatum* Sieb. Et Zucc. 15 g, *Urceola quintaretii* (Pierre) D. J. Middleton. 15 g, *Dendrocnide urentissima* (Gagnep.) Chew. 15 g, *Thyrocarpus sampsonii* Hance. 15 g, *Kadsura coccinea* (Lem.) A.C. Smith. 20 g, *Clematis chinensis* Osbeck. 20 g, *Acanthopanax gracilistylus* W.W.Smith. 15 g, *Semiliquidambar cathayensis* H. T. Chang. 15 g, *Aristolochia mollissima* Hance. 15 g, *Flemingia prostrata* Roxb. f. ex Roxb. 20 g, *Homalomena occulta* (Lour.) Schott. 20 g, *Caesalpinia sappan* L. 20 g, *Herba Artemisiae Anomalae*. 20 g, *Lycium chinense* Mill. 15 g, *Smilax china* L. 20 g, *Smilax glabra* Roxb. 20 g, *Curculigo orchioides* Gaertn. 15 g, *Drynaria fortunei* (Kunze) J.Sm. 15 g, Two of *Scolopendra subspinipes mutilans* L.Koch., *Buthus martensii* Karsch. 10 g, *Japalura polygonata* Hallowell. 10 g, Ten of *Pheretima aspergillum* (E.Perrier)., Twenty of *Pinus tabulaeformis* Carr.  *Schefflera arboricola* Hayata. 15 g, *Bauhinia championii* (Benth.) Benth. 20 g, *Carduus crispus* L. 20 g, *Inula cappa* (Buch. -Ham.) DC. 20 g, *Clematis chinensis* Osbeck. 20 g, Two of *Gekko gecko* Linnaeus., *Sarcandra glabra* (Thunb.) Nakai. 15 g, *Entada phaseoloides* (L.) Merr. 15 g, *Caesalpinia sappan* L. 20 g, *Herba Artemisiae Anomalae*. 20 g, *Ardisia crenata* Sims. 15 g, *Euonymus fortunei* (Turcz.) Hand. Mazz. 15 g, *Bungarus fasciatus*. 15 g, *Talinum paniculatum* (Jacq.) Gaertn. 20 g, *Cibotium barometz* (L.) J.Sm. 20 g, *Drynaria fortunei* (Kunze) J.Sm. 20 g, *Pheretima aspergillum* (E. Perrier). 15 g, *Uraria lagopodioides* (Linn.) Desv. ex DC. 15 g, *Arisaema franchetianum* Engl. 15 g, *Pinellia ternate* (Thunb.) Breit. 30 g.  *Talinum paniculatum* (Jacq.) Gaertn. 20 g, *Dioscorea Panthaicae* Rhizoma. 20 g, *Curculigo orchioides* Gaertn. 15 g, *Dimocarpus longan* Lour. 20 g, *Drynaria fortunei* (Kunze) J.Sm. 20 g, *Cibotium barometz* (L.) J.Sm. 20 g, *Panax notoginseng* (Burkill)F. H. Chen ex C.H. Chow. 10 g, *Pinus tabulieformis* Carr. 15 g, *Bauhinia championii* Benth. 20 g, *Kadsura coccinea* (Lem.) A.C.Smith. 20 g, *Gaultheria yunnanensis* (Franch.) Rehd. 20 g, *Ficus simplicissima* Lour. 20 g, *Clematis chinensis* Osbeck. 20 g, Two of *Pteris vittata* L., *Buthus martensii* Karsch. 10 g. | Zhu. (2005) |
| 4 | Zhuang Medicine Comprehensive Treatment | **Basic internal prescription:** *Bauhinia championii* (Benth.) Benth. 15 g, *Flemingia prostrata* Roxb. 30 g, *Rubus sieboldi* Blume.10 g, *Glechoma longituba* (Nakai) Kupr. 10 g, *Campanumoea javanica*Blume. 12 g, *Dioscorea op posita* Thunb. 15 g, *Massa Medicata* Fermentata. 10 g, *Parabarium micranthum* (A.DC.) Pier. 12 g, *Spatholobus suberectus* Dunn. 20 g, *Piper kadsura* (Choisy) 0hwi. 15 g, *Cinnamomum cassia* Presl. 15 g, *Glycyrrhiza uralensis* Fisch. 6 g. Severe upper limb disease plus *Notopterygium incisum* Ting ex H.T. Chang, *Clematis chinensis* Osbeck; severe lower limb disease plus *Achyranthes bidentata* Blume., *Angelica pubescens* Maxim.f. *biserrata* Shan et Yuan., *Chaenomeles speciosa* (Sweet) Nakai.; blood deficiency plus: *Rehmannia glutinosa* (Gaetn.) Libosch. ex Fisch. et Mey., *ligusticum chuanxiong* Hott., *Paeonia lactiflora* Pall.; yang deficiency plus *Psoralea corylifolia* L., *Eucommia ulmoides* Oliv.; blood stasis plus *Boswellia carterii* Birdw., *Commiphora myrrha* Engl.; the cold is added to *Aconitum carmichaelii* Debx. and *Asarum heterotropoides* Fr.Schmidt val. *Mandshuricum* (Maxim.) Kitag.; wet even add *Atractylodes lancea* (Thunb.) DC., *Poria cocos* (Schw.) Wolf., *Coix lacryma-jobi* L. var. *Mayuen* (Roman.) Stapf.; hot even add *Anemarrhena asphodeloides* Bge., *Trachelospermum jasminoides* (Lindl.) Lem.  **Ironing drug composition:** *Pittosporum balansae* DC. 50 g, *Zanthoxylum nitidum* (Roxb.) DC. 50 g, *Tinospora sinensis* (Lour.) Merr. 50 g, *Sarcandra glabra* (Thunb.) Nakai. 50 g, *Erythrina variegata* Linnaeus. 50 g, *Lycopodium japonicum* Thunb. 50 g. The above drugs were broken into pieces, packed in a bag, immersed in 1500-2000 ml water for 20 min, heated and decocted, and stopped heating 15 min after boiling. The bag was heated (to the extent that it could be adapted without ironing) for repeated ironing of the affected area for 20 min, and then the affected area was soaked with the above-drug water while hot.  **Zhuang medicine Xian dian zhi** | Dou et al. (2006) |
| 5 | Zhuang medicine orally and with drug bamboo jar therapy | **Internal use of Si Teng Tang:** *Embelia parviflora* Wall. 15 g, *Tinospora sinensis* (Lour.) Merr. 15 g, *Fissistigma polyanthum*. 15 g, *Fibraurea recisa* Pierre. 10 g, *Kadsura coccinea* (Lem.) A.C. Smith. 10 g, as the basic prescription. **Clinical differential diagnoses:** Yang syndrome plus *Zanthoxylum nitidum* (Roxb.) DC. 15 g, *Lonicera japonica* Thunb. 20 g, *Trachelospermum jasminoides* (Lindl.) Lem. 30 g; yin syndrome plus *Thalictrum omeiense* W.T.Wanget S. H.Wang. 30 g, *Sinomenium acuturn* (Thunb.) Rehd.et Wils. 30 g, *Toddalia asiatica* (L.) Lam. 15 g; in the deficiency syndrome: 30 g of *Millettia specisoa* Champ. and 20 g of *Parabarium micranthum* (A.DC.) Pier. were added, etc.  **Medicated bamboo jar therapy:** Put 40 g each of *Parabarium micranthum* (A.DC.) Pier., *Rhododendron molle* G.Don., *Alangium chinense* (Lour.) Harms., *Kadsura coccinea* (Lem.) A.C.Smith. and *Kadsura longipedunculata* Finetet Gagnep., 30 g of *Paederia scandens* (Lour.) Merr., 20 g each of *Tinospora sinensis* (Lour). Merr. and *Claoxylon indicum* (Reinw.ex Bl.) Hassk., wrap them in cloth, add 5000 ml of water and boil them, put them into a bamboo pot for about 20 min, remove the pot and cupping on the swollen and painful joint while it is hot, then take out the medicine bag and apply hot compress on the affected area, after that needle prick, then repeat cupping and hot compress on the same place for 1 time. | Zhong et al. (2008) |
| 6 | Teng huang lian tang/ Teng huang lian jiu | **Teng huang lian tang:** Damp-Heat Type: *Fibraurea tinctoria* Lour. 10 g, *Cyclea hypoglauca* (Schauer) Diels. 15 g, *Ilex pubescens* Hooker & Amott. 45 g, *Illigera trifoliata* (Griff.) Dunn. 15 g, *Mappianthus iodoides* Hand- Mazz. 30 g, *Sarcandra glabra* (Thunb.) Nakai. 30 g. Wind-cold-damp type: Teng huang lian tang plus *Iodes vitiginea* (Hance) Hemsl. 15 g, *Iodes vitiginea* (Hance) Hemsl. 30 g. Phlegm and dampness fetish blood type: Teng huang lian tang plus *Callerya dielsiana* var. dielsianaHarms. 15 g, *Aechynanathus austroyunna-nensis* W. T. Wang var. *guangxiensis* (Chun ex W. T. Wang) W. T. Wang. 12 g.  Teng huang lian tang: 1 dose of Teng huang lian Decoction, soaked in 1000 ml of Mi san hua Wine for 30 days. | Wu and Liu. (1999) |
| 7 | Chui feng teng tang/ Chui feng teng jiu | **Chui feng teng tang:** *Iodes vitiginea*（Hance）Hemsl. 30 g, *Fissistigma polyanthum* (Hook. f. & Thomson) Merr*.* 15 g、Red *Iodes vitiginea* (Hance) Hemsl. 24 g, Black *Iodes vitiginea* (Hance) Hemsl*.* 30 g, *Callerya dielsiana* var*. dielsianaHarms*. 12 g, *Artocarpus styraci folius* Pierre. 18 g, *Aristolochia fordiana* Hemsl. 6 g.  **Chui feng teng jiu:** Chui feng teng tang one dose with Mi san hua Wine 1000 ml, soaked 30 d, external rubbing the affected area, several times a day. | Wu and Liu. (2000) |
| 8 | Long zuan tong bi ke li | *Toddalia asiatica* (L.) Lam., *Kadsura coccinea* (Lem.) A.C. Smith., *Alangium chinense* (Lour.) Harms., *Zanthoxylum nitidum* (Roxb.) DC., *Sinomenium acutum* (Thunb.) Rehd.et Wils., *Bauhinia championii* (Benth.) Benth., *Spatholobus suberectus* Dunn., *Ficus hirta* Vahl. | Yao et al. (2022) |
| 9 | Zhuang Medicine Bai wei long zuan | *Toddalia asiatica* (L.) Lam., *Kadsura coccinea* (Lem.) A.C. Smith., *Zanthoxylum nitidum* (Roxb.) DC., *Alangium chinense* (Lour.) Harms., *Bauhinia championii* (Benth.) Benth., *Sinomenium acuturn* (Thunb.) Rehd.et Wils., *Bauhinia championii* (Benth.) Benth., *Ficus hirta*Vahl., *Spatholobus suberectus* Dunn. | Yao. (2022) |
| 10 | Qing du Shen jin tang | *Sarcandra glabra* (Thunb.) Nakai. 20 g, *lnicera japonica* Thunb. 20 g, *Ilex rotunda* ThLmb. 20 g, *Sinomenium acuturn* (Thunb.) Rehd.et Wils. 15 g, *Zanthoxylum nitidum* (Roxb.) DC. 1 g, *Spatholobus suberectus* Dunn. 15 g, *Lycopodium jaPonicum* Thunb. 15 g. | Li et al. (2020) |
| 11 | Zhuang medicine taken internally and combined with medicinal bamboo jar therapy | **Internal:** *Fissistigma polyanthum* (Hook. f. & Thomson) Merr., *Aralia cordata* Thunb., *Tinospora sinensis* (loureiro) Merrill., each 15 g, *Kadsura coccinea* (Lem.) A.C.Smith. 10 g, *Psychotria serpens* Linn. 10 g. (Basic prescription); the basic prescription of Yin syndrome adds 15 g of *Toddalia asiatica* (L.) Lam., 25 g of *Thalictrum omeiense* W.T.Wanget S. H.Wang. and 25 g of *Trachelospermum jasminoides* (Lindl) Lem.; the basic prescription of deficiency syndrome adds 20 g of *Parabarium micranthum* (A.DC.) Pier. and 25 g of *Millettia specisoa* Champ.  **Medicated bamboo jar therapy:** *Alangium chinense* (loureiro) Harms. 40 g, *Kadsura coccinea* (Lem.) A.C.Smith. 40 g, *Rhododendron molle* G.Don. 40 g, *Parabarium micranthum* (A.DC.) Pier. 40 g, *Kadsura longipedunculata* Finetet Gagnep. 40 g, *Claoxylon indicum* (Reinw.ex Bl.) Hassk. 25 g. | Huang. (2019) |
| **Uygur medicine** | | | |
| 1 | Xin jiang jia long dan / Xin jiang a pa si (Xin jiang Gentian, also known as Xin jiang Apas) | *Gentianella turkestanorum* (Gand.) Holub. | Zhou et al. (2017) |
| 2 | Chui guo teng / Ye xi gua / Lao shu gua / Ci shan gan (Ceylon Caper, also known as wild watermelon, mouse melon, prickly mountain citrus) | *Capparis zeylanica* Linn. |  |
| 3 | Uyghur medicine comprehensive therapy | **Tasteless mucous type:** (mature agent) *Vitis vinifera* L., *Ficus caricaLinn*., *Pimpinella anisum* L., *Foeniculum vuLgare* Mill., *Glycyrriza Uralensis* Fisch.G. Glabra L., *Rosa rugosa* Thunb. and other crude drugs made of 500ml mixture. **Clearing agent:** colchicine mixture. **Compound four root decoction mixture:** *Capparis spinosa* L., *Citrullus colocynthis* (L.) Schrad., *Ophiopogon japonicus* (L.f.) Ker-Gawl., *Colchicum autumnale* L., *Pimpinella anisum* L., *Apium graveolens* var*. Rapaceum*., *Prunus Dulcis* and other crude drugs made of 900 ml mixture.  **Gypsum-like mucilage type:** (Mature agent) *Adiantum capillus-veneris* L., *Alhagi pseudalhagi* Desv., *Nymphaea tetragona* Georgi., seedless *Vitis vinifera* L., *Rumex dentatus* L., *Lavandula angustifolia* Mill., *Euphorbia humifusa* Willd*.* ex Schltdl., *Ficus carica* Linn., *Rosa rugosa* Thunb. candy paste, *Viola tianshanica* Maxim. and other crude drugs made of 1000 ml mixture. **Cleaning agent:** *Adiantum capillus-veneris* L., *Cordia dichotoma* Forst. f., *Dracocephalum moldavica* L., *Glycyrriza Uralensis* Fisch.G.Glabra L., *Pimpinella anisum* L., *Rosa rugosa* Thunb., *Alhagi pseudalhagi* Desv., *Cuscuta chinensis* Lam., *Cassia angustifolia* Vahl., Seedless *Vitis vinifera* L., *Rumex dentatus* L., *Ziziphus jujuba* Mill., *Lavandula angustifolia* Mill., *Euphorbia humifusa* Willd*.* ex Schltdl., Ficus *carica* Linn., *Rosa rugosa* Thunb. candy paste, Qing xie *Cassia fistula* L., *Prunus Dulcis*. oil made 1000 ml mixture. Compound Si gen Decoction Mixture (Ibid.)  **Astringent balgham type:** (mature agent) *Adiantum capillus-veneris* L., *Cordia dichotoma* Forst., *Dracocephalum moldavica* L., *Glycyrriza Uralensis* Fisch.G.Glabra L., *Pimpinella anisum* L., *Rosa rugosa* Thunb., *Alhagi pseudalhagi* Desv., seedless *Vitis vinifera* L., *Rumex dentatus* L., *Ziziphus zizyphus*., *Lavandula angustifolia* Mill., *Euphorbia humifusa* Willd*.* ex Schltdl., *Ficus carica* Linn., *Rosa rugosa* Thunb. sugar paste made of 1000 ml mixture. **Cleaning agent:** *Adiantum capillus-veneris* L., *Cordia dichotoma* Forst. f*.,* *Dracocephalum moldavica* L., *Glycyrriza Uralensis* Fisch.G.Glabra L., *Pimpinella anisum* L., *Rosa rugosa* Thunb., *Alhagi pseudalhagi* Desv., *Cuscuta chinensis* Lam., *Cassia angustifolia* Vahl., seedless *Vitis vinifera* L., *Rumex dentatus* L., *Ziziphus zizyphus*., *Lavandula angustifolia* Mill., *Euphorbia humifusa* Willd*.* ex Schltdl., *Ficus carica* Linn., *Rosa rugosa* Thunb. sugar cream, *Cassia fistula* L., *Prunus Dulcis.* oil made of 400-600 ml mixture. Compound Sigen Decoction Mixture (Ibid.)  **Abnormal sinking liquid type:** (mature agent) *Cordia dichotoma* Forst. f., *Dracocephalum moldavica* L., *Glycyrriza Uralensis* Fisch.G.Glabra L., *Pimpinella anisum* L., *Adiantum capillus-veneris* L., *Rumex dentatus* L., *Ziziphus zizyphus*., *Lavandula angustifolia* Mill., *Euphorbia humifusa* Willd*.* ex Schltdl., thorn sugar water decoction to take. **Cleaning agent:** *Cordia dichotoma* Forst., *Dracocephalum moldavica* L., *Glycyrriza Uralensis* Fisch.G.Glabra L., *Pimpinella anisum* L., *Adiantum capillus-veneris* L., *Alhagi pseudalhagi* Desv., *Ziziphus zizyphus*., *Lavandula angustifolia* Mill., *Euphorbia humifusa* Willd*.* ex Schltdl., *Cuscuta chinensis* Lam., *Cassia angustifolia* Vahl., *Cassia fistula* Linn., *Prunus dulcis*. oil, *Rumex dentatus* L., *Terminalia chebula* Retz. water decoction to take. Compound Si gen Decoction Mixture (Ibid.) | Elyar. (2020) |
| **Dai medicine** | | | |
| 1 | Hei xin shu pi (Black-hearted bark) | *Cassia siamea* Lam. | Pan et al. (2019) |
| 2 | Ku teng (Tenacious Condorvine Root or Herb) | *Millettia dielsiana* Harms. |  |
| 3 | San ye man jing (vitex trifolia) | *Vitex trifolia* Linn. |  |
| 4 | Qing niu dan (Limacia Sagittata) | *Tinospora sagittata* (Oliv.) Gagnep. |  |
| 5 | La chang shu gen (Cassia fistula root) | *Cassia fistula* L. |  |
| 6 | Hei pi die da (Stem of Manyflower Fissistigma) | *Fissistigma polyanthum* (Hook. f. et Thoms.) Merr. |  |
| 7 | Bai hua chou mu dan (White flower Clerodendrum bungei) | *Clerodendrum chinense* var. simplex (Moldenke) S.L. Chen. |  |
| 8 | Da ye gou teng (largeleaf gambirplant branchlet) | *Uncaria macrophylla* Wall*.* in Roxb. |  |
| 9 | Zhu ye lan (Purpleback Murdannia) | *Arundina graminifolia* (D. Don) Hochr. |  |
| 10 | Ke teng / Guo gang long (Entada Sten / climbing entada) | *Entada phaseoloides* (Linn.) Merr. | Xu et al. (2021) |
| 11 | Ya long mei lan shen / Fang feng zhi tong fang | Ya sha ban / Chu feng cao *(Sambucus chlnensis* Lindl*)*, Li luo / Wen shu lan (*Crinum asiaticum* L. var*. sinicum* (Roxb. ex Herb.) Baker), Mo ha lang / Da bu gu dan (*Adhatoda uentricosa* (Wall.) Nees.), Mo ha hao / Ya zui hua (*Justicia adhatoda* L.), Ya jia re long / Da che qian cao (*Plantago major* L.), Bu lei / Ye jiang (*Zingiber cammuner* Roxb.), Hao ming / Huang jiang (*Curcuma longa* L.), Bao dong dain / Wei zi (*Baliospermum effusum* Pax et Hoffm.), the fresh products were 30-50 g each, cut and mashed, added with medicinal wine or rice water for super-heat standby. | Zhou et al. (2017) |
| 12 | Ya long meng sha hou prescription | **wind-fire syndrome:** *Tinospora sagittata* (Oliv.) Gagnep. 5-15 g, *Acorus calamus* L. 10-20 g, *Eclipta prostrata L*. 10-15 g, *Fissistigma polyanthum* (Hook. f. et Thoms.) Merr. 5-15 g, *Uncaria macrophylla* Wall. in Roxb. 5-15 g, *Cassia fistula* Linn. 5-15 g, *Cassia siamea* Lam. 5-15 g.  **Wind-cold symptoms:** *Kummerowia striata* (Thunb.) Schindl. 10-15 g, *Curcuma longa* L. 10-15 g, *Dioscorea Zingiberensis* C.H.Wright. 10-15 g, *Amomum maximum* Roxb. 10 g, *Vitex trifolia* L. 10 g, *Cassia fistula* Linn. 12-18 g. | Pan et al. (2018) |
| 13 | Ke luo lei xiao feng san | *Tinospora sagittata* (Oliv.) Gagnep., *Acorus calamus* L., *Eclipta prostrata* L., *Cassia siamea* Lam., *Celastrus paniculatus* Willd. | Pan et al. (2019) |
| 14 | Han man long chu zhi feng tong tang | *Paederia foetida* L., *Dioscorea Zingiberensis* C.H.Wright., *Curcuma longa* L., *Amomum maximum* Roxb., *Vitex trifolia* L. |  |
| **Miao medicine** | | | |
| 1 | Hong he ma (Laportea bulbifera) | *Laportea bulblfera* (Sieb.etZucc.) Wedd. | Tang et al. (2022) |
| 2 | Hei gu teng (Black bone rattan) | *Periploca forrestii* Schltr. | Zhu et al. (2020) |
| 3 | Fei long zhang xue (Radix Toddaliae Asiaticae) | *Toddalia asiatica* (L.) Lam. |  |
| 4 | Lei gong teng (Tripterygium wilfordii) | *Tripterygium wilfordii* Hook.f. |  |
| 5 | Qing feng teng (Caulis Sinomenii) | *Sinomenium acutum* (Thunb.) Rehd.et Wils. and *Sinomenium scutum* (Thunb.) Rehd.et Wils.var. *cinereum* Rehd.et Wils. |  |
| 6 | Zhui feng san (starflowerlike loosestrife root or herb) | *Lysimachia paridiformis* Franch*.var.Stenophylla* Franch. |  |
| 7 | Wei ling xian (chinese clematis root) | *Clematis chenesis* Osbeck. and *Clematis hexapstala* Pall. and *Clematis manshurica* Rupr. |  |
| 8 | Ba jiao feng (chinese alangium) | *Alangium chinense* (Lour.) Harms. |  |
| 9 | Jin tie suo (Psammosilenes Radix) | *Psammosilene tunicoides* W. C. Wu et C. Y. Wu. |  |
| 10 | Tie kuai zi (Tibetan Hellebore) | *Chimonanthus praecor* (L.) Link. and *Chimonanthus nitens* Oliv. |  |
| 11 | Dɑ xue teng (Stem of Sargentgloryvine) | *Sargentodoxa cuneata* (Oliv.) Rehd.et Wils. |  |
| 12 | Tou gu xiang (yunnan wintergreen stem and leaf) | *Gaultheria yunnanensis* (Franch.) Rehd． |  |
| 13 | Qi ye lian (Scandent Schefflera Stem and Leaf) | *Hepatica nobilis* var*. Obtusa*. |  |
| 14 | Kun ming shan hai tang (Tripterygium Hypoglaucum) | *Tripterygium hypoglaucum* (Devl.) Hutch. |  |
| 15 | Xi xian cao (Root of Common St. Paulswort) | *Siegesbeckia orientalis* L. |  |
| 16 | Nan she teng (Stem of Oriental Bittersweet) | *Celastrus orbiculatus* Thunb. |  |
| 17 | Jie gu cao (common codariocalyx branchlet and leaf) | *Sambucus chinensis* Lindl. |  |
| 18 | Bai hua dan (white flower lead word herb) | *Plumbago zeylanica* L. |  |
| 19 | Chou mu dan gen (Root of Rose Glorybower) | *Clerodendron bungei* Steud. |  |
| 20 | Niu wei cai (riparian greenbrier root and rhizome) | *Smilax riparia* A. DC. |  |
| 21 | Mu gua (papaya) | *Chaenomeles speciosa* (Sweet) Nakai. |  |
| 22 | Jiang Huang (tumeric) | *Curcuma longa* L. |  |
| 23 | Hei long gu (Forrest Silkvine) | *Periploca forrestii* Schltr. | Liu et al. (2018) |
| 24 | Wu Xianɡ Xue Tenɡ (longpeduncle kadsura stem) | *Clematis fasciculiflora* Franch. | Li. (2020) |
| 25 | Yan zhi (Artocarpus tonkinensis) | *Artocarpus tonkinensis* A. Chev. ex Gagnep. | Adorisio et al. (2019) |
| 26 | Jin wu jian gu fang | *Cibotium barometz* (L.) J. Sm. 15 g, *Homalomena occulta* (Lour.) Schott. 10 g, *Periploca forrestii* Schltr. 10 g, *Zaocys dhumnades* (Cantor). 10 g, *Sabia parviflora* Wall. ex Roxb. 15 g, *Panax notoginseng* (Burkill) F.H. Chen ex C.H. Chow. 3 g, *Paeonia tacti lora* Pall. 15 g, *Curcuma longa* L. 15 g, *Glycyrrhiza uralensis* Fisch. 3 g. | Yao et al. (2017) |
| 27 | Jin wu jian gu decoction | *Cibotium barometz* (L.) J.Sm. 15 g, *Aconitum kusnezoffii* Reichb. 10 g (Fried first), *Periploca forrestii* Schltr. 15 g, *Homalomena occulta* (Lour.) Schott. 15 g, *Sinomenium acuturn* (Thunb.) Rehd.et Wils. 30 g, *Curcuma longa* L. 20 g, *Paeonia lactiflora* Pall. 30 g, *Panax notoginseng* (Burkill) F.H. Chen ex C.H. Chow. 3 g (taking medicine after infusion). | Ma et al. (2010) |
| 28 | Hei gu teng zhui feng huo luo capsule | *Sinomenium acuturn* (Thunb.) Rehd.et Wils., *Periploca forrestii* Schltr., *Lysimachia paridiformis* Franch.var*. stenophylla* Franch. | Gu et al. (2018) |
| 29 | Si da xue Prescription | *Spatholobus suberectus* Dunn., *Mezoneuron cucullatum* (Roxb.) Wight & Arn., *Gardneria angustifolia* Wall.in Roxb., *Sargentodoxa cuneata* (Oliv.) Rehd.et Wils. | Zhu et al. (2020) |
| 30 | Tou gu xiang fang | *Gaultheria yunnanensis* (Franch.) Rehd. 20 g, *Toddalia asiatica* (L.) Lam. 15 g, *Periploca forrestii* Schltr. 15 g, *Chimonanthus praecox* (L.) Link. 15 g, *Sargentodoxa cuneata* (01iv.) Rehd. et Wils. 20 g, *Ampelopsis delavayana* Planch. 20 g, *Mucuna sempervirens* Hemsl. 20 g, *Epimedium acuminatum* Franch. 20 g, *Cocculus orbiculatus* (L.) DC. 15 g. | Guo. (2015) |
| 31 | Wen bi decoction prescription | *Aconitum carmichaeli* Debx. 10 g, *Zingiber officinale* Rosc. 20 g, *Cinnamomum cassia* Presl. 12 g, *Epimedium brevicornu* Maxim. 15 g, *Astragalus membranaceus* (Fisch.) Bunge. 20 g, *Angelica sinensis* (Oliv.) Diels. 12 g, *Rehmannia glutinosa* (Gaertn.) DC. 15 g, *Atractulodes macrocephala* Koidz. 10 g, *Coix lacryma-jobi* L.var. *mayuen* (Roman.) Stapf. 30 g, *Scolopendra subspinipes mutilans* L.Koch. 5 g, *Cynanchum panniculatum* (Bge.) Kitag. 15 g. | Xiang et al. (2018) |
| 32 | Bone and joint pain prescription | *Kadsura Longipedunculata* Finetet Gagn., *Laportea bulblfera* (Sieb.etZucc.) Wedd., *Schisandra sphenanthera* Rehd. et Wils. | Wu et al. (2022) |
| 33 | Miao medicine fumigation prescription | Ga ba hao hu (*Tripterygium wilfordii* Hook. f.), Sa dou nao li (*Sinomenium acuturn* (Thunb.) Rehd.et Wils.), Ja jiong zhen gong you (*Schisandra sphenanthera* Rehd. et Wils.), Ga gong bu suo xue ga ba (*Toddalia asiatica* (L.) Lam.), Bao jia li you (*Aconitum kusnezoffii* Reichb.), Jia dou gei zong (*Clematis chinensis* Osbeck.), Dou wo gei jia fei you (*Taxillus chinensis* (DC.) Danser.), Jia e xi (*Epimedium brevicornu* Maxim.), Wo xiang qin (*Ligusticum chuanxiong* Hort.), *Notopterygium incisum* Ting ex H.T. Chang., *Cinnamomum cassia* Presl., *Asarum heterotropoides* Fr.Schmidt val. *Mandshuricum* (Maxim.) Kitag., etc. | Luo. (2016) |
| 34 | Wu teng san external application | *Sargentodoxa cuneata* (Oliv.) Rehd.et Wils. 20 g, *Spatholobus suberectus* Dunn. 20 g, *Sinomenium acuturn* (Thunb.) Rehd.et Wils. 20 g, *Tripterygium wilfordii* Hook. f. 20 g, *Periploca forrestii* Schltr. 20 g, *Ephedra sinica* Stapf. 20 g, *Ligusticum chuanxiong* Hort. 20 g, *Cinnamomum cassia* Presl. 20 g, *Prunus persica* (L.) Batsch. 20 g, *Boswellia carterii* Birdw. 20 g, *Bombyx mori* Linnaeus. 20 g. | Huang et al. (2012) |
| **Yi medicine** | | | |
| 1 | Jin tie suo (Tuniclike Psammosilene Root) | *Psammosilene tunicoides* W.C. Wu et CY. Wu. | Deng et al. (2018) |
| 2 | Yi medicine self-prepared prescription | **Basic prescription:** *Althaea rosea* (L.) Cav. 20 g, *Piper puberulum* (Benth) Maxim. 20 g, *Colquhounia coc cinea* Wall.var*.mollis* (Schlecht.) Prain. 15 g, *Dioscorea collettii* Hook.f. 20 g, Bian di (*Heteropogon contortus* (L.) Beauv.ex Roem.et Schult.) 10 g.  Cold-dampness obstruction type, basic prescription adds *Aconitum carmichaelii* Debx. Orally; for damp-heat stagnation type, basic prescription adds *Anemarrhena asphodeloides* Bge. and Jiao huang bai (*Phellodendron amu-rense* Rupr.) orally; for liver-kidney deficiency type, basic prescription adds *Eucommia ulmoides* Oliv. and *Achyranthes bidentata* Blume. Orally. | Long and Zhou. (2011) |
| **Yao medicine** | | | |
| 1 | Jiu long zuan (Stem of Champion Bauhinia) | *Bauhinia championii* (Benth) Benth. | Tang et al. (2022) |
| 2 | Da zuan (Kadsura coccinea) | *Kadsura coccinea* (Lem.) A.C.Smith. |  |
| 3 | Da hong zuan (Kadsura heteroclita) | *Kadsura heteroclite* (Roxb.) Craib. |  |
| 4 | Xiao zuan (Schiandra Sphenanthera) | *Kadsura longipedunculata* Finetet Gagnep. |  |
| 5 | Si fang zuan (Creeping treebine) | *Cissus hastata* (Miq.) Planch. |  |
| 6 | Bing lang zuan (Sargentgloryvine Stem) | *Sargentodoxa cuneata* (01iv.) Rehd.et Wils. |  |
| 7 | Di zuan (Philippine Flemingia Root) | *Flemingia philippinensis* Merr. et Rolfe. |  |
| 8 | Pang tong medicinal bath | *Uncaria rhynchophylla* (Miq.) Miq.ex Havil. 30 g, *Liquidambar formosana* Hance. 25 g, *Acanthopanar gracilistulus* W.W.Smith. 25 g, *Parabarium huaitingii* Chun et Tsiang. 30 g, *Cissus hastata* (Miq.) Planch. 25 g, *Toddalia asiatica* (L.) Lam. 30 g, *Lycopodium japonicum* Thunb. 30 g, *Achyranthes bidentata* BL. 30 g, *Tinospora sinensis* (loureiro) Merrill. 30 g, *Zanthoxylum nitidum* (Roxb.) DC. 15 g, *Taxillus chinensis* (DC.) Danser. 25 g, *Eucommia ulmoides* Oliv. 25 g, add an appropriate amount of water, and boil for 1h. Remove the dregs of a decoction put the medicinal juice into a bathtub and add an appropriate amount of Mi san hua wine while it is still hot. | Liu et al. (2020) |
| **Tu jia medicine** | | | |
| 1 | San bai bang (Cochinchinese Asparagus Root) | *Asparagus cochinchinensis* (Lour.) Merr. | Nan et al. (2019) |
| 2 | Fei long zhang xue (Asiatic Toddalia Root) | *Toddalia asiatica* (L.) Lam. |  |
| 3 | Hei lao hu (Blacktiger Kadsura) | *Kadsura coccinea* (Lem.) A.C.Smith. | Yang et al. (2021) |
| 4 | Yi xing nan wu wei zi (Kadsura heteroclita) | *Kadsura heteroclita* (Roxb.) Craib. | Yu et al. (2019) |
| 5 | Fu fang zhu jie shen pian | *Panar jaPonicus* C.A. Mey., *Epimedium brevicornu* Maxim., *Paeonia lactiflora* Pall., *Dioscorea nipponica* Makino. | Tan. (2011) |
| 6 | Qing jiang rheumatic medicated wine | *Hylomecon Japonicum* (Thunb.) Prantl., *Diphylleia sinensis* H.L.Li., *Acanthopanax giraldii* Harms., *Cynanchum panniculatum* (Bge.) Kitag., *Cocculusorbiculatus* (Linnaeus) Candolle., *Gynura segetum* (Lour.) Merr., *Toddaliaasiatica* (L.) Lam., *Cynoglossum zeylanicum* (Vahl) Thunb.ex Lehm., *Panar jaPonicus* C.A. Mey., *Euphorbia hylonoma* Hand.Mazz., *Aralia elata* (Miq.) Seem., *Gynura segetum* (Lour.) Merr., *Dioscorea nipponica* Makino. | Jiang. (2007) |
| **Dong medicine** | | | |
| 1 | Fu Zheng Tong Luo wan combined with fire acupuncture tonic and diarrhea method | **Internal:**  **Fu Zheng Tong Luo wan:** *Diphasiastrum complanatum* (L.) Holub., *Hedera Nepalensis* k.koch var. *Sinensis* (Tobl.) Rehd., *Dysosma versipellis* (Hance) M. Cheng ex T. S. Ying., *Ilex pubescens* Hook. et Arn., *Cynanchum panniculatum* (Bge.) Kitag., *Whitmania Pigra* Whitman., *Ilex cornuta* Lindl.ex Paxt., *Cassytha filiformis* L., *Schisandra chinensis* (Turcz.) Baill., *Hippocampus kelloggi* Jordan et Snyder. (Drugs drying, grinding into fine powder, water flooding into pills)  Fire Acupuncture Therapy of Dong Medicine | Su. (2016) |
| 2 | Song zhang fang | *Usnea diffracta* Vain. 20g, *Polygonum cuspidatum* Sieb. Et Zucc. 30g. | An et al. (2018) |
| **She medicine** | | | |
| 1 | Long xu teng (Bauhinia championii) | *Bauhinia championii* (Benth.) Benth. | Xu et al. (2018) |

**Notes:**

1. Due to the limitation of the national language, the Latin name of the base plant cannot be found for some of the ethnic medicines, so the original Hanyu Pinyin translation is retained to ensure its accuracy.
2. If there is a Latin name of its original plant in Chinese and English articles, directly adopt the Latin name in the article; If there is no Latin name of the basic source plant, it will be searched from the clinical diagnosis and treatment knowledge base (http://lczl.med.wanfangdata.com.cn/) and the basic information base of Chinese herbal medicine-Yaozhi data (https://db.yaozh.com/zhongyaocai) according to the name in the article. When there is more than one base plant in the medicinal plant, this paper will choose the Latin name of the base plant that is ranked as the first one in the database.
3. The second column in the table is composed of Chinese pinyin and English names of herbs; The prescription consists of Chinese pinyin.

**Reference**

Adorisio, S., Fierabracci, A., Muscari, I., Liberati, A.M., Calvitti, M., Cossignani, L., et al. (2019). Artocarpus tonkinensis Protects Mice Against Collagen-Induced Arthritis and Decreases Th17 Cell Function. *Front Pharmacol*. doi:10, 503. 10.3389/fphar.2019.00503

An, Y., Huang, Y., Cao, Y.P., An, Y., Huang, Y., Cao, Y.P., et al. (2018). Clinical efficacy of Dong medicine Song zhang fang on rheumatoid arthritis patients in active stage. *J Guizhou Univ Tradit Chin Med.* 40(1), 87-89,100. doi:10.16588/j.cnki.issn1002-1108.2018.01.022

Bai, P., Xin, S.S., and Dong, Y. (2015). Anti-inflammatory effect of Mongolian medicine compound Sendeng-4 on adjuvant arthritis in rats. *J Beijing Univ Tradit Chin Med.* 38(03), 186-189+219.

Bao, F.R. (2003). Mongolian medicine treatment of rheumatoid arthritis 100 cases. *China's Naturopathy.* (11), 57-58. doi:10.19621/j.cnki.11-3555/r.2003.11.092

Chen, X.O., Luo-Song, T.X., Si-Lang, G.S., and Zhao, G. (2021). Effect of Tibetan medicine Mai xie therapy on 90 patients with rheumatoid arthritis and serum cytokines. *J Basic Chin Med.* 27(06), 990-994. doi:10.19945/j.cnki.issn.1006-3250.2021.06.025

Cao, Z.Y., Zhang, Q.H., and Pang, Y.Z. (2022). Influence of Zhuang medicine total alkaloids of Qingfengtang on the expression of serum P-selectin, PECAM-1 in rat models suffering from rheumatoid arthritis. *West J Tradit Chin Med.* 35(08), 23-25.

Dan, Z. (2009). Clinical observation on 100 cases of rheumatoid arthritis treated with Tibetan medicine Shi wei ru xiang San. *J Med Pharm Chin Minorities.* 15(05), 10. doi:10.16041/j.cnki. cn15-1175.2009.05.001

Dai, J.L. (2007). Mongolian medicine rheumatism I capsule plus bath therapy in the treatment of rheumatoid arthritis curative effect observation of 48 cases. *J Med Pharm Chin Minorities.* (08), 20-21. doi:10.16041/j.cnki.cn15-1175.2007.08.013

Dou, X.B., Li, F.Z., and Tan, S.C. (2006). Zhuang medicine comprehensive treatment of 37 cases of rheumatoid arthritis. *Guangxi J Tradit Chin Med.* (02), 45-46.

Deng, Y., Qian, Z.G., Liu, W.L., Ding, X., and Chen, H.F. (2018). Anti-inflammatory activity of Psammosilenes Radix and total saponins in vivo. *Chin J Exp Tradit Med Formulae.* 24 (07), 165-170. doi:10.13422/j.cnki.syfjx.20180633

Elyar Y. (2020). Effect of traditional Uyghur medicine comprehensive therapy on quality of life in patients with rheumatoid arthritis. *Xinjiang Med Univ*. 2020. doi:10.27433/d.cnki.gxyku.2020. 000827

Gesang, C.R., Wei, Y.F., Bai, W.T., Liu, C., Hou, M., Xie, Y.H., et al. (2017). Pharmacological action of Tibetan medicine caulis Tinosporae Sinensis against collagen-induced rheumatoid arthritis in rats. *Tradit. Chin. Drug Res. Clin. Pharmacol.* 28(03), 327-331. doi:10.19378/j.iss n.1003-9783.2017.03.013

Ga, R.M. (2004). Treatment of 210 cases of rheumatoid arthritis with Tibetan medicine Sang dang Nie e. *J Med Pharm Chin Minorities*. (01), 9. doi:10.16041/j.cnki.cn15-1175.2004.01.007

Ga, R.M. (2008). Treatment of 220 cases of rheumatoid arthritis with the Tibetan medicine Zhen wu su jiao. *J Med Pharm Chin Minorities*. (01), 13. doi:10.16041/j.cnki.cn15-1175.2008.01.0 09

Gu, J.H., Wu, Y.L., Chen, W.L., and Zhao, P. (2018). Clinical efficacy and mechanism of Heiguteng Zhuifeng Huoluo Capsule in treating rheumatoid arthritis. *Chin J Exp Tradit Med Formulae.* 24(03), 180-184. doi:10.13422/j.cnki.syfjx.2018030180

Guo, C.Y. (2015). Research on the threrapy of rheumatoid arthritis by Miaonational medicine compound Touguxiang decoction. *Gui Yang: Gui Yang college Tradit Chin Med*. 2015. doi:10.7666/d.Y3066015

Huang, Y. (2009). Studies of Chemical constituents and screening for anti-RA active fraction of Myricaria germanica(L.) Desv. *Qinghai Norm Univ*. 2009.

He, Q., Tan, X., Geng, S., Du, Q., Pei, Z., Zhang, Y., et al. (2022). Network analysis combined with pharmacological evaluation strategy to reveal the mechanism of Tibetan medicine Wuwei Shexiang pills in treating rheumatoid arthritis. *Front Pharmacol*. 13, 941013. doi:10.3 389/fphar.2022.941013

Huang, X.J., Wang, J., Muhammad, A., Tong, H.Y., Wang, D.G., Li, J., et al. (2021). Systems pharmacology-based dissection of mechanisms of Tibetan medicinal compound Ruteng as an effective treatment for collagen-induced arthritis rats. *J Ethnopharmacol*. 272, 113953. doi:10.1016/j.jep.2021.113953

Hai, X.Z., and Dai, C.H. (1996). Mongolian medicine treatment of rheumatoid arthritis 40 cases. *J Med Pharm Chin Minorities.* (03), 18.

Huang, Z.G. (2019). Briefly discussing the clinical efficacy of Zhuang medicine taken internally plus Zhuang medicine material bamboo jar therapy in the treatment of rheumatoid arthritis. *World Latest Med Inf.* 19(83), 202+206. doi:10.19613/j.cnki.1671-3141.2019.83.127

Huang, Y, Ma, W.K., Liu, L.M., Yao, X.M., Wang, Y., Zhou, J., et al. (2012). A randomized controlled clinical study of Miao Yao Wu Teng San external application combined with western medicine in the treatment of acute phase of the acute period cold-wet resistance collaterals type rheumatoid arthritis. *J Practical Tradit Chin Internal Med.* 26(14), 1-2+12.

Jin, R.Y. (2020). Clinical observation on the treatment of rheumatoid arthritis in 100 cases with the Tibetan medicine Er shi wu wei Lv xue Pill. *Psychological Mon.* 15(07), 176. doi:10.19 738/j.cnki.psy.2020.07.160

Jiang, J.L. (2007). Experimental and clinical study on the treatment of rheumatoid arthritis with Qingjiang rheumatic medicine wine. *Hubei Univ Chin Med*. 2007.

Lu, Y.Q., and Zhang, Y.Z. (2010). Clinical observation of 70 cases of rheumatoid arthritis treated with Ru Yi Zhen Bao Pill. *Chin Mod Med. 17*(18), 88-89.

Li, Z., Nie, L., Li, Y., Yang, L., Jin, L., Du, B., et al. (2022). Traditional Tibetan Medicine Twenty-Five Wei'er Tea Pills Ameliorate Rheumatoid Arthritis Based on Chemical Crosstalk Between Gut Microbiota and the Host. *Front Pharmacol.* 13, 828920. doi:10.3389/fphar.2022. 828920

Liu, W., Wu, Y.H., Hu, S.Y., Zhong, C.L., Gao, M.L., Liu, D.W., et al. (2016). A multicenter, randomized, double-blind, placebo-controlled trial evaluating the efficacy and safety of Tong Luo Hua Shi capsule, a modernized Tibetan medicine, in patients with rheumatoid arthritis. *Trials*. 17, 359. doi:10.1186/s13063-016-1481-3

Liu, J., Dong, Q.M., Hao, H., Wu, H., Jia, L.F., He, B. (2020). Study on intestinal immune mechanisms of Hezi (Terminalia chebula) extract in CIA model rats. *Chin Arch Tradit Chin Med*. 38 (10), 35-39 + 263-264. doi:10.13193/j.issn.1673-7717.2020.10.008

Liu, Y. (2011). The study on anti-inflammatory compound Mongolian medicine of its screening and effects. *Inn Mong Agric Univ*. 2011.

Li, N., Long, Z.Y., Liang, Y., Teng, C.F., Huang, A.S., Li, F.Z. (2020). Clinical observation on the treatment of rheumatoid arthritis with Zhuang medicine Qing du Shen jin Decoction. *J Med Pharm Chin Minorities.* 26(12), 1-3. doi:10.16041/j.cnki.cn15-1175.2020.12.026

Liu, T., Wang, X., He, Y.L., Wang, Y., Dong, L., Ma, X., et al. (2018). In Vivo and In Vitro Anti-Arthritic Effects of Cardenolide-Rich and Caffeoylquinic Acid-Rich Fractions of Periploca forrestii. *Molecules.* 23(8), 1988. doi:10.3390/molecules23081988

Li, X. (2020). Miao medicine Wu xiang xue teng on serum IL-23 and IL-17 in rats with rheumatoid arthritis. *Chin Health Care Nutr.* 30(32), 68-69.

Luo, J.X. (2016). Clinical observation on the treatment of rheumatoid arthritis by fumigation with Miao medicine. *Yiyao Qianyan.* 6(31), 348-349.

Long, Q.S., and Zhou, S.C. (2011). Clinical observation on the treatment of rheumatoid arthritis with Yi medicine self-prepared prescription. *J Changchun Univ Chin Med.* 27(06), 1011-1012. doi:10.13463/j.cnki.cczyy.2011.06.008

Liu, L., Qin, Q., Xu, L., Li, T., Li, Y.Y., Zhang, M. (2020). Efficacy of Yao medicine Pangtong medicinal bath for treating rheumatoid arthritis. *Guangxi Med J.* 42(13), 1635-1638.

Ma, Q.F. (2001). Clinical observation on the treatment of 30 cases of rheumatoid arthritis with the Chinese and Tibetan medicine Shi wei meng peng powder. *J Chin High Alt Med Biol*. (02), 47.

Ma, D.B. (2009). Clinical observation on 153 cases of rheumatoid arthritis treated with Tibetan medicine Wu Wei Gan Lu Liquid. *J Med Pharm Chin Minorities.* 15(04), 15-16. doi:10.1604 1/j.cnki.cn15-1175.2009.04.009

Ma, D.B. (1998). Clinical observation on 120 cases of rheumatoid arthritis treated with Er shi wu wei Lv xue Pill of Tibetan medicine. *J Med Pharm Chin Minorities*. (03), 14.

Ma, W.K., Zhong, Q., Yao, X.M., Tang, F., Huang, Y., An, Y., et al. (2010). Effect of the Jinwu Jiangu decoction for rheumatoid arthritis with methotrexate resistance. *J Tradit Chin Med*. 51(11), 993-995. doi:10.13288/j.11-2166/r.2010.11.044

Ma, C.Y., and Chen, W.C. (2005). Chinese Hui Medicine. *Yin Chuan: Ning Xia People's Publishing House*. 2005, 128.

Naren G.R.L. (2019). Observation on the curative effect of Mongolian medicine combined with medicated bath in the treatment of rheumatoid arthritis and Mongolian medicine nursing. *J Med Pharm Chin Minorities.* 25(11), 79-80. doi:10.16041/j.cnki.cn15-1175.2019.11.041

Na, R., Su, R.G., Du, L., Lan, H., and BurenjiRi G.L. (2020). Clinical study on the treatment of rheumatoid arthritis with traditional Mongolian medicine. *J Med Pharm Chin Minorities.* 26(07), 34-37. doi:10.16041/j.cnki.cn15-1175.2020.07.021

Naren, M.D.L. (2011). Clinical Observation of 105 Cases of Rheumatoid Arthritis Treated by Mongolian Medicine Bath. J Med Pharm Chin Minorities. 17(08), 11. doi:10.16041/j.cnki.cn 15-1175.2011.08.019

Niu, Y. (2010). Research on Hui Hui Medicine Formula. *Yin chuan: Sunshine Publishing House,* 2010.

Nan, Y.Y., Lin, H.J., Hao, R.X., and Yuan, L. (2019). Overview of rheumatoid arthritis treatment by Tujia medicine. *China's Naturopathy.* 27(06), 5-7. doi:10.19621/j.cnki.11-3555/r.2019.0604

Pan, L.W., Ding, Z.L., Zhao, G.G., Yu, C.M., Duan, L.S., Li, H.W., et al. (2019). Research status of Dai medicines for treatment of rheumatoid arthritis. *Chin J Inf on Tradit Chin Med.* 26 (02), 137-140.

Pan, L.W., Ding, Z.L., Zhao, G.G., Yu, C.M., Duan, L.S., Li, H.W., et al. (2018). Clinical observation on the Curative effect of Yalongmengshahou prescription treatment of rheumatoid arthritis. *Acta Chin Med.* 33(06), 1107-1110. doi:10.16368/j.issn.1674-8999.2018. 06.263

Qi, F.S. (2014). Clinical efficacy of Mongolian medicine Bie chong zhao na in the treatment of rheumatoid arthritis. *J Med Pharm Chin Minorities.* 20(04), 12. doi:10.16041/j.cnki.cn15-117 5.2014.04.036

Qi, H.G.J.L.T., Chen, Q.Q.G., and Cao, Q.M.G. (2003). Mongolian medicine Tuolei-15 treatment of rheumatoid arthritis in 30 cases. *China's Naturopathy*. (07), 54. doi:10.19621/j.cnki.11-35 55/r.2003.07.084

Qi, X.M. (2011). Mongolian medicine combined with medicated bath in the treatment of 106 cases of rheumatoid arthritis. *Chin J Ethnomedicine Ethnopharmacy.* 20(24), 7.

Shen P. (2002). Study on the anti-rheumatoid arthritis effect and mechanism of Tibetan medicine Pterocarpus pterocarpus. *Chengdu Univ Tradit Chin Med.* 2002.

Shan, Y.D. (2005). The Herbal Medicine of Hui. *Yin chuan: Ning xia People's Publishing House*. 2005.

Su, W.C. (2016). Clinical study on the treatment of rheumatoid arthritis by Dong medicine fire acupuncture tonic and diarrhea method combined with Dong medicine Fu Zheng Tong Luo Wan. *J Med Pharm Chin Minorities.* 22(03), 6-8. doi:10.16041/j.cnki.cn15-1175.2016.03.004

Tang, J., Zhang, Q., Wu, T., Chen, S.Y., Chen, Y., Li, Y.T., et al. (2022). Potential pharmacodynamic substances of Laportea bulbifera in treatment of rheumatoid arthritis based on serum pharmacochemistry and pharmacology. *Chin J Chin Mater Med.* 47(17), 4755-4764. doi:10.19540/j.cnki.cjcmm.20220609.201

Tang, B.L., Qin, L., Mo, D.D., Zhou, S.L., Hou, S.L., Cheng, Y. (2022). Research progress of traditional Yao drug Zuan herbs against rheumatoid arthritis. *Guangxi Med J.* 44(01), 101-104.

Tan, Q.L. (2011). The effects of anti-inflammatory of compound Panax Japonicus Tablets in rheumatoid arthritis mice. *Chin Med Her.* 8(28), 27-28.

Tang, J., Zhang, Q., Wu, T., Chen, S.Y., Chen, Y., Li, Y.T., et al. (2022). Potential pharmacodynamic substances of Laportea bulbifera in treatment of rheumatoid arthritis based on serum pharmacochemistry and pharmacology. *Chin J Chin Mater Med.* 47(17), 4755-4764. doi:10.19540/j.cnki.cjcmm.20220609.201

Wang, Y.J., Zhong, X.Y., Wang, X.H., Zhong, Y.H., Liu, L., Liu, F.Y., et al. (2022). Activity of Codonopsis canescens against rheumatoid arthritis based on TLRs/MAPKs/NF-κB signaling pathway and its mechanism. *Chin J Chin Mater Med.* 47(22), 6164-6174. doi:10.19540/j.c nki.cjcmm.20220727.401

Wan-Ma C.D. (1999). Observation of 78 cases of rheumatoid arthritis treated with Tibetan medicine in internal and external baths. *J Med and Pharm Chin Minorities.* (01), 15-16.

Wang, S., Du, Q., Sun, J., Geng, S., and Zhang, Y. (2022). Investigation of the mechanism of Isobavachalcone in treating rheumatoid arthritis through a combination strategy of network pharmacology and experimental verification. *J Ethnopharmacol.* 294, 115342. doi:10.1016/j. jep.2022.115342

Wu, F.Y. (2010). Mongolian medicine treatment of rheumatoid arthritis 118 cases. *J Med Pharm Chin Minorities.* 16(05), 13. doi:10.16041/j.cnki.cn15-1175.2010.05.012

Wange, R.Y. (2007). Clinical Observation of 67 Cases of Rheumatoid Arthritis Treated with Mongolian Medicine. *J Med Pharm Chin Minorities.* (12), 16. doi:10.16041/j.cnk i.cn15-117 5.2007.12.010

Wu, Z.D., and Liu, Y.H. (1999). Zhuang medicine Teng huang lian Decoction for the Treatment of 40 Cases of Rheumatoid Arthritis. *Chin J Ethnomedicine Ethnopharmacy.* (03), 133-135.

Wu, Z.D., and Liu, Y.H. (2000). Clinical report on 568 cases of rheumatic cold arthralgia treated with Zhuang medicine blowing wind vine soup and blowing wind vine wine. [C]//Professional Committee on Rheumatic Diseases of the Chinese Society of Integrative Medicine. Compilation of papers from the Fourth National Academic Conference on Combined Chinese and Western Medicine and Rheumatic Diseases. *Guangxi Litang Agric Orthopaedic Hospital*. 2000, 91-92.

Wu, C.L., Yang, J.Q., Long, D.Y., and Zhang, S.Y. (2022). Clinical effect observation on rheumatoid arthritis treated of prescription by traditionnal Miao medicine. *J Med Pharm Chin Minorities*. 28(02), 1-3. doi:10.16041/j.cnki.cn15-1175.2022.02.036

Xiao-Wu, Y.B. (2017). Clinical study on the treatment of rheumatic arthritis with Tibetan medicine Wuwei Lezhe Decoction Powder. *Biped Health.* 26(13), 167+171. doi:10.19589/j.cnki.issn10 04-6569.2017.13.095

Xian, B. (2020). Study on the clinical effectiveness of Tibetan medicine Wu Wei Gan Lu bath therapy for rheumatoid arthritis. *Cardiovascular Dis Electronic J Integr Tradit Chin West Med*. 8(30), 160+166. doi:10.16282/j.cnki.cn11-9336/r.2020.30.118

Xian, D.M., and Buren, J.R.G.L. (2006). Treatment of 97 cases of chronic joint pain with five-flower medicinal baths in Mongolian medicine. *Chin J Ethnomedicine Ethnopharmacy.* (04), 222.

Xu, J.H., Luo, M., Jiang, H.Q., Xiong, H., Mei, Z.N., Yang, G.Z. (2021). Therapeutic effect of Entada phaseoloides on bovine type II collagen-induced rheumatoid arthritis in rats. *J South-Cent Minzu Univ (Natural Science Edition).* 40(01), 32-38.

Xiang, Y.X., Long, D.Y., and Guo, W.W. (2018). Clinical efficacy evaluation of Wenbi yin in the treatment 20 cases with rheumatoid arthritis in cold-damp Bi resistance type. *J Med Pharm Chin Minorities.* 24(3), 7-9. doi:10.16041/j.cnki.cn15-1175.2018.03.006

Xu, W., Liu, X.F., Zhen, H.Y., Xu, S.H., Lan, Z.J., Huang, Z.H., et al. (2018). Anti-rheumatoid arthritis basic research and development and utilization of Fujian national medicine Bauhinia championii. *Fujian Province, Fujian Univ Tradit Chin Med*. 2018-12-01.

Yangben Z.X. (2016). External application of Tibetan medicine in the treatment of rheumatoid arthritis. *J Med Pharm Chin Minorities.* 22(08), 43-44. doi:10.16041/j.cnki.cn15-1175.2016.0 8.030

Yang, S.S., Song, X.L., Gan, Y.Y., Chen, H.H., Shi, J.N., Liu, X.D. (2022). effect of Geranium wilfordii cataplasm on rheumatoid arthritis in rats. *Drug Eval Res.* 45(09), 1816-1821.

Yao, L., Cheng, S., Yang, J., Xiang, F., Zhou, Z., Zhang, Q., et al. (2022). Metabolomics reveals the intervention effect of Zhuang medicine Longzuantongbi granules on a collagen-induced arthritis rat model by using UPLC-MS/MS. *J Ethnopharmacol*. 294, 115325. doi:10.1016/j.j ep.2022.115325

Yao, L. (2022). Chemical composition of Zhuang medicine Longzuantongbi granules and Metabolomic study to reveal the intervention effect on rheumatoid arthritis rats. *Minzu Univ Chin.* 2022*.* doi:10.27667/d.cnki.gzymu.2022.000025

Yao, X.M., Ning, Q.Y., Hou, L., Li, D.X., Zhou, J., Ma, W.K., et al. (2017). Antiinflammatory effects of Miao medicine Jinwujiangu decoction on synovial cells in human with rheumatoid arthritis. *Chin J Hosp Pharm.* 37(12), 1134-1138. doi:10.13286/j.cnki.chinhosppharmacyj.20 17.12.04

Yang, Y.P., Jian, Y.Q., Liu, Y.B., Ismail, M., Xie, Q.L., Yu, H.H., et al. (2021). Triterpenoids From Kadsura coccinea With Their Anti-inflammatory and Inhibited Proliferation of Rheumatoid Arthritis-Fibroblastoid Synovial Cells Activities. *Front Chem*. 9, 808870. doi: 10.3389/fchem.2021.808870

Yu, H.H., Lin, Y., Zeng, R., Li, X., Zhang, T., Tasneem, S., et al. (2019). Analgesic and anti-inflammatory effects and molecular mechanisms of Kadsura heteroclita stems, an anti-arthritic Chinese Tujia ethnomedicinal herb. *J Ethnopharmacol*. 238, 111902. doi:10.10 16/j.jep.2019.111902

Zhao, L., Ye, J., Wu, G.T., Peng, X.J., Xia, P.F., Ren, Y. (2015). Gentiopicroside prevents interleukin-1 beta induced inflammation response in rat articular chondrocyte. *J Ethnopharmacol.* 172, 100-107. doi:10.1016/j.jep.2015.06.031

Zhou, L., Wang, R.Z., Zhao, S.P., Tao, L., Hao, F.S., Ma, Y., et al. (2021). Analgesic effects of ethno-medicine cynanchum komarovii on chronic inflammatory pain. *J Diseases Monitor Control.* 15(06), 421-423+426. doi:10.19891/j.issn1673-9388.(2021)06-0421-04

Zhu, Q.M. (2005). Zhuang medicine and Zhuang prescriptions for rheumatic and rheumatoid arthritis.[C]//Chinese Society of Ethnic Medicine, Guang xi District Department of Health, Guang xi College of Traditional Chinese Medicine. 2005 National First Zhuang Medicine Academic Conference and National Ethnic Medicine Experience Exchange Conference Compilation of Papers. *Cenxi Daye Jimintang Pharm*. 2005, 226-227.

Zhong, L.Y., Li, F.Z., and Xie, A.Z. (2008). 36 cases of rheumatoid arthritis treated with Zhuang medicine and bamboo jar therapy. *Guangxi J Tradit Chin Med.* (03), 51-52.

Zhou, X.P., Han, L., Ye, Y.Y., Wang, R.Z., Zhang, Y.M., Bai, C.C. (2017). Progress on treatment of rheumatoid arthritis with ethnodrugs. *Chin J Chin Mater Med.* 42(12), 2398-2407. doi:10.195 40/j.cnki.cjcmm.2017.0115

Zhu, C.H., Zhou, X., and Chen, H.G. (2020). Advances on treatment of rheumatoid arthritis with Miao medicine. *Chin J Mod Applied Pharm.* 37(21), 2669-2677. doi:10.13748/j.cnki.issn10 07-7693.2020.21.020

**Supplementary Table 2.**

The characteristics of pharmacology, toxicology and clinical/intervention research involved in ethnic medicine in this paper.

| 1 | Authentic |
| --- | --- |
| 2 | Reliable source |
| 3 | Widely used |
| 4 | Well characterised |
| 5 | Active ingredients known |
| 6 | The chemical profile of the active ingredients/marker compounds is characterised qualitatively and quantitatively |
| 7 | Free of adulteration and contamination |
| 8 | Consistent |
| 9 | Batch to batch variation is limited |
| 10 | Stable |
